# Cas9/Nickase-induced allelic conversion by homologous chromosome-templated repair in *Drosophila* somatic cells

**DOI:** 10.1126/sciadv.abo0721

**Published:** 2022-07-01

**Authors:** Sitara Roy, Sara Sanz Juste, Marketta Sneider, Ankush Auradkar, Carissa Klanseck, Zhiqian Li, Alison Henrique Ferreira Julio, Victor Lopez del Amo, Ethan Bier, Annabel Guichard

**Affiliations:** 1 Section of Cell and Developmental Biology, University of California, San Diego, 9500 Gilman Drive, La Jolla, CA 92093-0335, USA.; 2 Instituto de Ciências Biomédicas (ICB), Universidade Federal do Rio de Janeiro, Av. Carlos Chagas Filho 373, Ilha do Fundão, Rio de Janeiro, 21941-902 RJ, Brazil.; 3 Tata Institute for Genetics and Society–UCSD, La Jolla, CA 92093-0335, USA.

## Abstract

Repair of double-strand breaks (DSBs) in somatic cells is primarily accomplished by error-prone nonhomologous end joining and less frequently by precise homology-directed repair preferentially using the sister chromatid as a template. Here, a *Drosophila* system performs efficient somatic repair of both DSBs and single-strand breaks (SSBs) using intact sequences from the homologous chromosome in a process we refer to as homologous chromosome-templated repair (HTR). Unexpectedly, HTR-mediated allelic conversion at the *white* locus was more efficient (40 to 65%) in response to SSBs induced by Cas9-derived nickases D10A or H840A than to DSBs induced by fully active Cas9 (20 to 30%). Repair phenotypes elicited by Nickase versus Cas9 differ in both developmental timing (late versus early stages, respectively) and the production of undesired mutagenic events (rare versus frequent). Nickase-mediated HTR represents an efficient and unanticipated mechanism for allelic correction, with far-reaching potential applications in the field of gene editing.

## Teaser

Efficient homolog-templated gene conversion is induced by a single nick in Drosophila somatic cells.

## INTRODUCTION

CRISPR (clustered regularly interspaced short palindromic repeat) components from *Streptococcus thermophilus* were initially found in bacteria as a defense system against phage infection and invasion by foreign DNA (*1*). Components of this natural immunity pathway have been modified and repurposed to produce specific and effective DNA cleavage and subsequent gene editing in eukaryotic cells for a myriad of applications in basic research, medicine, biotechnology, and agriculture (*2*–*7*). The Cas9 endonuclease, when paired with a chimeric guide RNA (single guide RNA or gRNA), cleaves DNA at a precise genomic site defined by the gRNA sequence. Resulting double-strand breaks (DSBs) then can be repaired by the cellular machinery through several pathways, which can be divided into two major groups: the error-prone nonhomologous end joining (NHEJ) pathway, which operates to reconnect loose ends, and the more precise homology-directed repair (HDR) pathways, which use a homologous DNA template for directional gene conversion events (*8*, *9*). Thus, random mutations can be created at specific sites through NHEJ, while for other applications, a donor plasmid with cargo DNA flanked by homology arms can direct insertion of desired sequences at the site of cleavage through HDR processes (*3*, *10*).

CRISPR components have also been configured into so-called active genetic systems to favor inheritance of desired traits and potentially modify insect, mammalian, and other populations *(**11*–*13**)*. Such gene-drive systems consist of DNA cassettes encoding a gRNA targeting their exact site of insertion. In the germ line of heterozygous animals, site-specific cleavage of the naïve chromosome results in copying of the gene-drive cassette onto the recipient chromosome, leading to its super-Mendelian transmission. As a consequence, “gene-drives” have the ability to spread rapidly through a targeted population and can be used to modify or suppress insect vectors (*13*, *14*).

A variation on the gene-drive principle termed “allelic-drive” includes an additional gRNA that targets a second locus, distinct from the site of gene-drive insertion (*15*, *16*). Such a gRNA can be designed in an allele-specific fashion, to promote dissemination of a cut-resistant allele at the expense of a cut-sensitive allele at the same site. A proof of principle for this strategy was conducted in *Drosophila* for the *Notch* locus (*15*). When combined with a Cas9-expressing transgene, the allelic-drive produced a high rate of allelic conversion resulting in super-Mendelian inheritance of the cut-resistant allele. This study also proposed a similar and equally efficient approach referred to as “copy grafting” that favors transmission of an allelic variant located near (rather than at) a cut-resistant site.

Both gene-drives and allelic-drives rely on copying of genetic material from the homologous chromosome following Cas9-induced position-specific cleavage. This HDR-mediated DNA repair process is highly efficient in the germ line but has generally been considered inefficient in somatic tissues (*17*). In a recent study, we challenged this premise by demonstrating that interhomolog copying of multikilobase gene cassettes can take place with great efficacy in somatic cells of *Drosophila* (*18*). These transgenic cassettes referred to as CopyCatchers were designed to reveal such somatic gene conversion events at several different loci. Upon targeted Cas9-dependent cleavage, CopyCatchers copied onto receiver chromosomes at unexpectedly high frequencies (30 to 50%).This process of Cas9-elicited interhomolog repair could also be demonstrated in human cells (albeit with lower efficiency) and in mouse embryos with increased dosage of Rad51 (*18*, *19*).

In the present study, we first provide an in-depth analysis of somatic homologous chromosome-templated repair (HTR) of mutant alleles of the *white* locus in *Drosophila*. The diverse combination of alleles tested revealed successful repair outcomes by HDR, NHEJ, or by combinations of these events through the production of red pigmented cell clones in an otherwise white-eyed mutant background. Next, we make the unexpected discovery that Cas9 nickase variants D10A and H840A, which generate targeted single-strand breaks (SSBs) rather than DSBs, also elicit HTR, and do so at levels yet higher than those achieved by Cas9. As expected (*20*), D10A and H840A induced very few NHEJ mutations. These Cas9 and Nickase-elicited HTR strategies rely on introduction of few genetic components and harness the cellular machinery to revert a genetic alteration to a wild-type functional state using endogenous templates. HTR-based approaches may enable the development of alternative gene therapies for correcting dominant or trans-heterozygous disease-causing DNA alterations.

## RESULTS

### Allele-specific DNA cleavage induces both HDR and NHEJ repair events

**Fig. 1.**
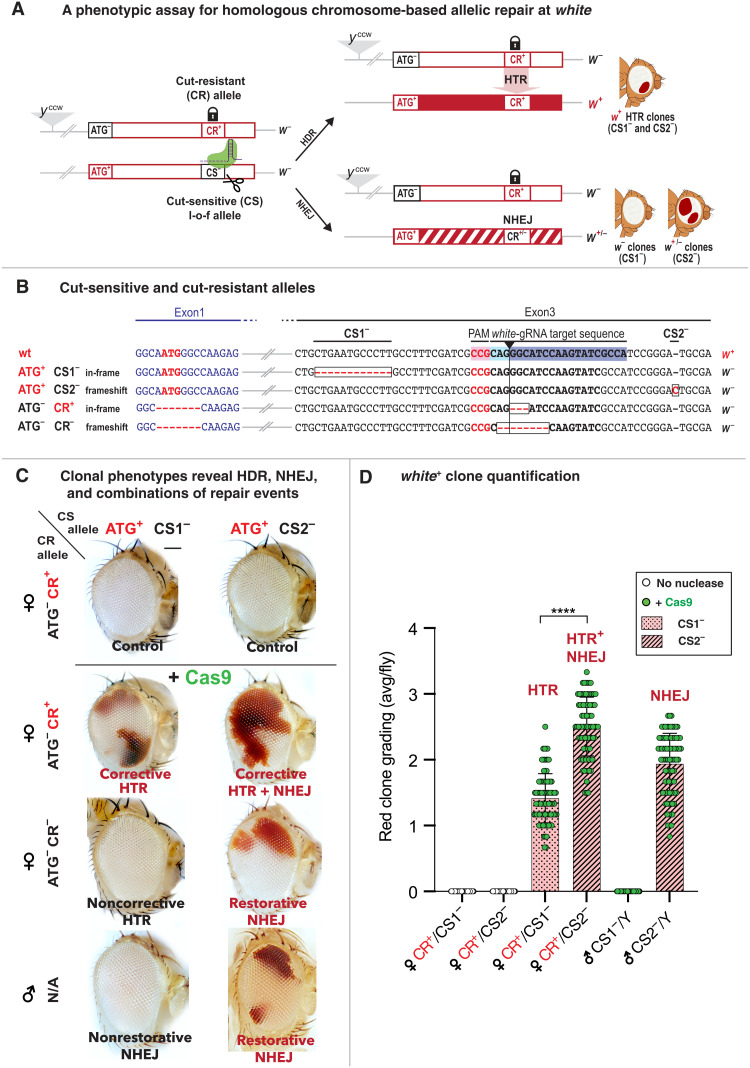
Somatic HDR and NHEJ repair following targeted DSB at *w* are differentially revealed by *w*^+^ **clones in *Drosophila*.** (**A**) Cas9-induced allelic correction at *w*. The *y*^ccw^ element encodes the *white*-gRNA relevant to this study, targeting cleavage of cut-sensitive (CS) *w*^−^ alleles. Functional cut-resistant allele (CR^+^) combined with an ATG^−^ mutation (~3.7-kb upstream) on the homologous chromosome may serve as repair template, to generate functional ATG^+^ CR^+^ combinations, producing *w*^+^ clones. The CS2^−^ (but not CS1^−^) allele can also be repaired through frame-restoring NHEJ. (**B**) Sequences of CS and CR alleles. gRNA binding site (blue), PAM site (pink), in-frame CS1^−^ (12-nt deletion), and frameshift CS2^−^ (1-nt insertion) alleles are indicated. The functional CR^+^ allele and l-o-f CR^−^ allele (3- and 8-nt deletions at the cut site, respectively) are combined with an ATG^−^ mutation. (**C**) Cas9-induced clonal phenotypes reveal HTR, NHEJ, and combination events. Control flies lacking Cas9 show no repair. *y*^ccw^ *w*^ATG− CR+^/*y*^+^ *w*^ATG+ CS1−^; vasaCas9 females show large solid red clones (corrective HTR). Cognate CR^−^ females (*y*^ccw^ *w*^ATG− CR−/ATG+ CS1−^, noncorrective HTR) show no red clones. *y*^ccw^ *w*^ATG+ CS1−^/Y; Cas9 males lacking a homologous X chromosome display no red clones (nonrestorative NHEJ), while CS2^−^ cognates show prevalent red clones (frame-restorative NHEJ). CR^+^/CS2^−^ females show more *w*^+^ clones (resulting from both NHEJ and HTR) than CS1^−^ cognates. CR^−^/CS2^−^ females (restorative NHEJ) show fewer red clones (resulting only from NHEJ-dependent frame restoration) . Scale bar, 100 μm. (**D**) Quantification of HTR and NHEJ events. Cas9-dependent repair is higher for CS2^−^ (HTR + NHEJ) than for CS1^−^ females (HTR only). *y*^ccw^ *w*^ATG+ CS1−^/Y; Cas9 males show no repair, while *y*^ccw^ *w*^ATG+ CS2−^/Y; Cas9 males show high-level repair (NHEJ-dependent frame restoration). *P* values for unpaired parametric *t* test analysis: ****P < 0.0001. Bars represent mean value and SD.

In the current study, we evaluate interhomolog allelic-conversion at the X-linked *white* (*w*) locus in somatic cells. We designed a variety of configurations in which allele-specific DNA cleavage leads to phenotypically visible and quantifiable repair events resulting in restoration of red eye pigmentation attributable to either HDR or NHEJ pathways (Fig. 1A). A previously validated transgenic *y*^ccw^ gene-drive element inserted in the *yellow* (*y*) locus produces a gRNA (*white*-gRNA) targeting cleavage in the third exon of the *w* gene (located ~2.4 Mb or ~1.5 centimorgans, from *y* toward the centromere) and is used throughout this study [Fig. 1A and (*21*)].

In addition, we created a set of “test” *w*^−^ alleles, with specific features allowing repair events to be visualized through the production of red (*w*^+^) eye clones in a *w*^−^ background. These loss-of-function (l-o-f) *w*^−^ mutations were created through coinjection of either of two gRNA-expressing constructs (5′ or 3′ gRNA) with a transient source of Cas9 into *w*^+^ embryos. These *w*^−^ test mutations lie in close proximity (25 to 30 nt) either 5′ or 3′ to the *white*-gRNA cut site (Fig. 1B and fig. S1) but remain sensitive to DNA cleavage [therefore termed cut-sensitive (CS)] and may be repaired either through HDR or NHEJ processes. Two alleles, CS1^−^ (an in-frame 12-nt deletion) and CS2^−^ (a 1-nt frame-shift insertion), were selected to conduct experiments described below (Fig. 1B). A second set of mutations was created by combining the *y*^ccw^ element and a source of Cas9 in *w*^+/+^ flies. DNA cleavage at *w* on both chromosomes, followed by NHEJ repair generated either functional or nonfunctional cut-resistant (CR^+^ and CR^−^) alleles recovered in the F2 generation (see Fig. 1B and fig. S2 for all CR alleles recovered). Chromosomes carrying these mutations act as protected “donors,” since they are no longer sensitive to DNA cleavage by Cas9 but can provide homologous templates for repairing cleaved cognate alleles. Last, we generated a *w*^−^ mutation deleting the ATG translation initiation site, which lies ~3.7 kb upstream from the *white*-gRNA cut site (Fig. 1B). This ATG^−^ mutation was combined with either of the cut-resistant CR^+^ or CR^−^ mutations and the *y*^ccw^ insertion (Fig. 1B) using Cas9-mediated allelic conversion. These latter combinations result in overall *w*^−^ donor chromosomes (*y*^ccw^ *w*^ATG− CR+^ and *y*^ccw^ *w*^ATG− CR−^) to permit scoring of repair events that are expected to result in *w*^+^ clones in an otherwise *w*^−^ background (Fig. 1A).

We visualized Cas9-driven repair of the CS1^−^ allele by crossing *w*^ATG+ CS1−^/Y; *vasaCas9* males [providing Cas9 ubiquitously in somatic cells (*22*)] with *y*^ccw^ *w*^ATG− CR+^ females. F1 female progeny consistently displayed large patches of red ommatidia of varying sizes (see Fig. 1, C and D, and fig. S3 for a range of graded phenotypes). These Cas9-dependent phenotypes indicate that DNA cleavage at the *white-*gRNA cut site occurred efficiently, presumably resulting in conversion of CS1^−^ to CR^+^ and copying the CR^+^ functional allele onto the homolog chromosome with the intact ATG^+^ start codon. We confirmed that these repair events were produced through HTR by examining eyes from *y*^ccw^ *w*^ATG− CR−^/*w*^ATG+ CS1−^; *vasaCas9*/+ F1 females, in which the cut-resistant donor allele was nonfunctional (CR^−^). As expected, such animals had entirely white eyes (non-corrective HDR; Fig. 1C). Since the outcome of the repair process for CS1^−^ depends exclusively on the nature of the CR donor allele (CR^+^: red clones; CR^−^: absence of red clones), we conclude that HTR is the exclusive process operating to produce *w*^+^ clones when the donor allele is functional (CR^+^). We also examined the progeny of *y*^ccw^ *w*^ATG− CR+^/*w*^ATG+ CS^ females and observed high frequencies of *w*^+^ individuals (~40%, close to the maximal theoretical 50% value), indicating that Cas9-dependent allelic conversion also was operating efficiently in the female germ line to produce functional ATG^+^ CR^+^ alleles (fig. S4).

### Isolating Cas9-induced NHEJ events

To probe the activity of the NHEJ pathway in our system, we produced males in which the *y*^ccw^ insertion was combined with a CS^−^ allele in the presence of Cas9. In such hemizygous animals lacking a second X chromosome, only NHEJ-based repair can operate following DNA cleavage. We found that NHEJ-based repair occurred in these animals but that repair phenotypes depended on which CS^−^ allele was tested. While males carrying the CS1^−^ allele had entirely white eyes, cognate CS2^−^ animals exhibited numerous red clones (Fig. 1C, bottom). Because the CS2^−^ allele is a frameshift mutation consisting of a 1-nt insertion in proximity to the *white-*gRNA cut site, we hypothesized that a fraction of NHEJ mutagenic events might restore the proper reading frame, potentially leading to functional *w*^+^ clones (frame-restorative NHEJ). In contrast, the CS1^−^ allele is an in-frame 12-nt deletion eliminating four essential amino acids. Such mutation is not amenable to functional restoration through mutagenic events produced by the NHEJ pathway, consistent with all flies displaying solid white eyes (Fig. 1, C and D). We confirmed this “frame restoration” hypothesis by sequencing individual *w*^+^ and *w*^−^ F2 progeny from *y*^ccw^ *w*^ATG+ CS2−^/Y; Cas9/+ males. *w*^+^ F2 animals (representing about a third of the total progeny) consistently revealed DNA lesions restoring the correct reading frame, while lesions found in their *w*^−^ siblings did not (fig. S5).

**Fig. 2.**
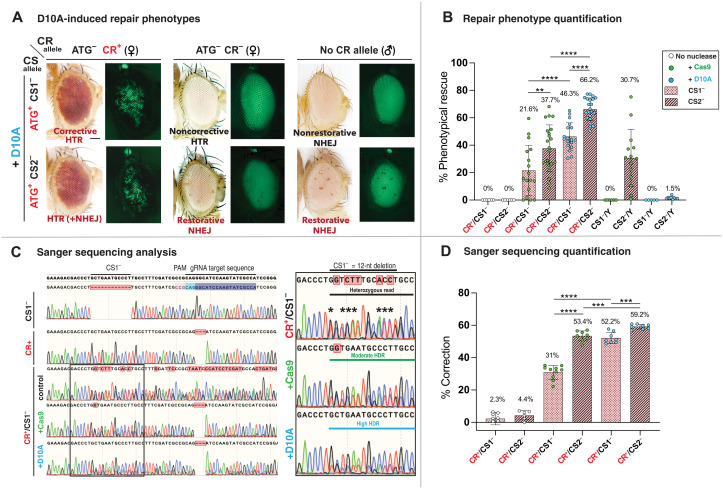
D10A elicit high-level HTR revealed by a dense array of small red clones and sequence analysis. (**A**) Clonal eye phenotypes following D10A-induced nicks, in flies carrying the *w* cut-sensitive ATG^+^ CS1^−^ (top row) or ATG^+^ CS2^−^ alleles (bottom row), combined with the *y*^ccw^ element and functional cut-resistant ATG^−^ CR^+^ *w^−^* allele (left), the nonfunctional ATG^−^ CR^−^ allele (middle), or lacking donor chromosome (males, right). Restored *w*^**+**^ function appears as a dense array of small pigmented *w*^+^ clones blocking GFP fluorescence (visible in *w*^−^ uncorrected areas) provided by the vasaCas9 insertion. In females, D10A-induced HTR corrects CS1^−^ and CS2^−^ using CR^+^ sequences (corrective HTR) but not CR^−^ sequences (noncorrective HTR). Low-level NHEJ-dependent repair of CS2^−^ in females produces small *w*^+^ clones (frame-restorative NHEJ, bottom middle). Similarly, CS2^−^/Y males display infrequent restorative NHEJ (bottom-right images), while CS1^−^ cannot be corrected (nonrestorative NHEJ, top-right images). (**B**) Global image quantification of Cas9- versus D10A-induced allelic repair. Total pigmented surface area quantified by ImageJ. The percentage correction is calculated as (pigmented area) × 100/(total surface of eye). The D10A-induced repair is significantly higher than that of Cas9 for both CS1^−^ (46% versus 22%) and CS2^−^ alleles (66% versus 38%). Repair of the CS2^−^ allele (38%) is higher than for the CS1^−^ allele (22%). In males, frequent NHEJ-only repair of CS2^−^ (~31%) is observed in Cas9 males, contrasting with low levels (1.5%) in CS2^−^/Y; D10A males. (**C**) DNA sequence chromatograms showing correction of CS1^−^ allele. The reference *w*^+^ DNA sequence near *white-*gRNA is shown on top. The inset on the right shows enlargement for analysis in (D) (quantified peaks indicated by asterisks). (**D**) Quantitative estimation of HTR of CS1^−^ to CR^+^ by Sanger sequencing. Correction percentages calculated for three to five individuals (two reads per individual), using marked double peaks. ****P < 0.0001, ***P < 0.001 and **P < 0.01.

In summary, allele-specific DNA cleavage at *w* leads to visible and quantifiable red eye clones that can be attributed specifically either to HDR exploiting using cut-resistant sequences from the homologous chromosome as a repair template, a process we refer to as HTR (in CR^+^/CS1^−^; Cas9/+ females), or to NHEJ (in CS2^−^*/*Y; Cas9/+ males). In addition, in females carrying the CS2^−^ allele (Fig. 1C, top right), both HDR- and NHEJ-driven repair can lead to formation of *w*^+^ clones. Consistent with this latter inference, the incidence of *w*^+^ clones for the CS2^−^ allele was significantly higher than for the CS1^−^ allele, in which such *w*^+^ clones result exclusively from HDR (Figs. 1D and 2B). We note that the additional gRNA (*y*-gRNA) expressed by the *y*^ccw^ construct targeting the *yellow* locus (used for initial insertion of this construct, see Materials and Methods) does not exert a significant influence on the repair processes at *w* (fig. S6).

### The D10A nickase supports efficient allelic conversion

Cas9D10A nickase (abbreviated as D10A hereafter), which lacks activity of the RuvC catalytic domain (*3*), cuts only the targeted DNA strand (the strand hybridizing to the gRNA) to generate single SSBs. Nickases have been used successfully for gene editing in mammalian cells, using exogenous DNA repair templates. Although typically less efficient than Cas9 for such gene editing, Nickases generate far fewer mutagenic events (*20*). We therefore wondered whether SSB could also promote HTR of a targeted allele. As in experiments described above, we produced *y*^ccw^ *w*^ATG− CR+^/*w*^ATG+ CS1−^; D10A/+ females. Unexpectedly, these individuals manifested strong repair phenotypes, in which most of the eye surface appeared pigmented (Fig. 2A, top left). In contrast, *y*^ccw^ *w*^ATG− CR−^/*w*^ATG+ CS1−^; D10A/+ females carrying the nonfunctional CR^−^ donor allele displayed entirely white eyes (top middle) and only rare minute *w*^+^ clones for *y*^ccw^ *w*^ATG− CR−^/*w*^ATG+ CS2−^; D10A/+ animals (bottom middle), consistent with the expectation that the cleavage-resistant allele present on the homologous chromosome serves as the repair template. Unlike Cas9-induced *w*^+^ clones presented in Fig. 1, which appeared as solid pigmented sectors of varying sizes and shapes, D10A-induced clones were small and distributed uniformly in a high-density salt-and-pepper pattern across the surface of the eye for both CS1^−^ and CS2^−^ alleles (Fig. 2A, left).

The contrasting patterns of *w*^+^ clones generated by intact Cas9 versus D10A nickase suggest that D10A-elicited DNA repair occurs later during development and with potentially greater efficiency relative to Cas9. Examination of these mosaic eyes under a fluorescence microscope revealed the pattern of DNA repair with superior cellular resolution than could be achieved with bright-field illumination, since green fluorescent protein (GFP) fluorescence (resulting from eye-specific expression of the 3XP3-GFP marker for the vasaD10A insertion) is readily visible in *w^−^* areas but is absorbed by eye pigments in *w*^+^ clones (fig. S7). Whereas Cas9-generated clones appear as large solid black sectors, D10A-generated phenotypes appear as a dense array of small black clones scattered throughout the entire surface of the eye (Fig. 2A, left, and fig. S7). The later developmental window for the generation of successful HDR events elicited by D10A versus Cas9 cannot be attributed to different expression profiles since both nucleases are expressed under control of the same *vasa* promoter. Rather, the differing clonal rescue patterns most likely reflect distinct mechanisms and/or timing of the repair process.

### The D10A nickase induces few NHEJ events

DNA nicks can be readily repaired by ligation and rarely create NHEJ-mediated lesions. We tested whether D10A had any mutagenic activity in our system by using males, in which the absence of a homologous X chromosome results only in NHEJ repair being capable of restoring *w*^**+**^ function in response to D10A-induced nicks for the CS2^−^ allele. We did visualize *w*^+^ clones in CS2^−^/Y; D10A males, which—contrasting with Cas9-induced repair phenotypes—were very small and rare (amounting to 8 to 25 small clones per eye), leaving most of the surface white (Fig. 2A, bottom right). In contrast, no *w*^**+**^ clones were produced in y^ccw^ *w*^ATG+ CS1−^/Y; D10A animals (Fig. 2A, top right), consistent with the observation that the CS1^−^ allele cannot be restored to functionality by NHEJ mutagenic events (Fig. 1, C and D).

### D10A is more efficient than Cas9 in inducing allelic correction

Because of their contrasting patterns (large clonal sectors versus scattered small clones), Cas9- and D10A-induced HDR phenotypes are difficult to compare quantitatively. We resolved this difficulty using an image-based quantification method, in which analysis of multiple eye pictures acquired in the GFP channel allows global quantitation of pigmented areas and estimation of the overall repair percentage. This analysis revealed that D10A-induced SSB leads to a significantly greater percentage of correction (~46%) than Cas9 (~22%) with regard to the CS1^−^ allele, for which only HDR events are able to restore the *w*^**+**^ function (Fig. 2B). D10A-elicited nicking of the CS2^−^ allele gave rise to an even higher percentage of repair (~66%). This difference with the CS1^−^ allele cannot be attributed solely to a contribution of NHEJ repair, which only amounted to a low 1.5% repair in *w*^ATG+ CS2−^/Y; D10A males (Fig. 2B). We conclude that differences in the position (5′ versus 3′ relative to the cut site) and the nature (1-nt versus 12-nt alterations) between the CS1^−^ and CS2^−^ alleles influence repair outcomes. Further analysis will be required to establish how the position and nature of the corrected allele affect the efficiency of HTR.

### Confirmatory molecular analysis of Cas9 versus D10A editing events

We complemented our phenotypic assessment of allelic repair events by genomic sequence analysis of regions encompassing the *white*-gRNA cleavage site in individual flies where DNA cleavage was produced either by Cas9 or D10A. DNA sequence chromatograms from control heterozygous flies (CR^+^/CS1^−^) revealed the expected overlapping peaks of similar heights, starting precisely at the CS1^−^ 12-nt deletion break point. In the presence of Cas9, peaks corresponding to the donor (CR^+^) sequences appeared consistently higher (Fig. 2C), at the expense of the receiver sequences (CS1^−^). Quantitative analysis of sequences from 6 to 10 independent reads revealed that correction increased from an average of ~2% in control flies to ~30% in Cas9-expressing flies, consistent with the pigmentation analysis (Fig. 2B). In D10A-expressing flies, this enhancement was even more pronounced, amounting to an average of ~52% correction, again indicating that repair following SSB results in more frequent HTR than observed for Cas9-induced DSB (Fig. 2D and Table 1). With regard to phenotypic quantification, correction of the CS2^−^ allele was even more efficient than for CS1^−^, amounting to an average of 53% for Cas9 and 59% for D10A. The 3-nt deletion at the cleavage site present in the donor allele also dominated in chromatograms from CR^+^/CS1^−^; D10A individuals, as is expected from a high proportion of HDR events. This feature was challenging to discern in CR^+^/CS1^−^; Cas9 animals, in which abundant NHEJ events presumably concealed this effect. These results also demonstrate that HTR occurs throughout the developing body and is not restricted to the eye tissue. In contrast to these results in somatic tissues, we found that D10A was not inducing efficient HTR in the germ line, suggesting that specific somatic factors/processes may determine the success of Nickase-induced HTR (fig. S8).

**Table 1.** Comparative summary of allelic repair induced by Cas9 and D10A. Key aspects of repair are listed as follows: type of breaks, somatic and germline HTR, somatic NHEJ, developmental timing of repair, multikilobase insertion copying, repair after bi-allelic cleavage, and pairing-independent repair.

**Nuclease: Type** **of cleavage**	**Somatic HTR**	**Germline repair** **visible in F2**	**NHEJ**	**Developmental** **timing**	**Multikilobase** **insertion** **copying**	**Repair after** **bi-allelic** **cleavage**	**Pairing-** **independent** **repair**
**Cas9: DSB**	Yes (moderate)	High levels	High levels	Early (large clones)	Yes (moderate)	No or very little	Not detected
**D10A: SSB**	Yes (efficient)	Very low levels	Very low levels	Late (small clones)	Low levels	Yes (efficient)	Yes (moderate)

### Comparative analysis of D10A and H840A repair outcomes

**Fig. 3.**
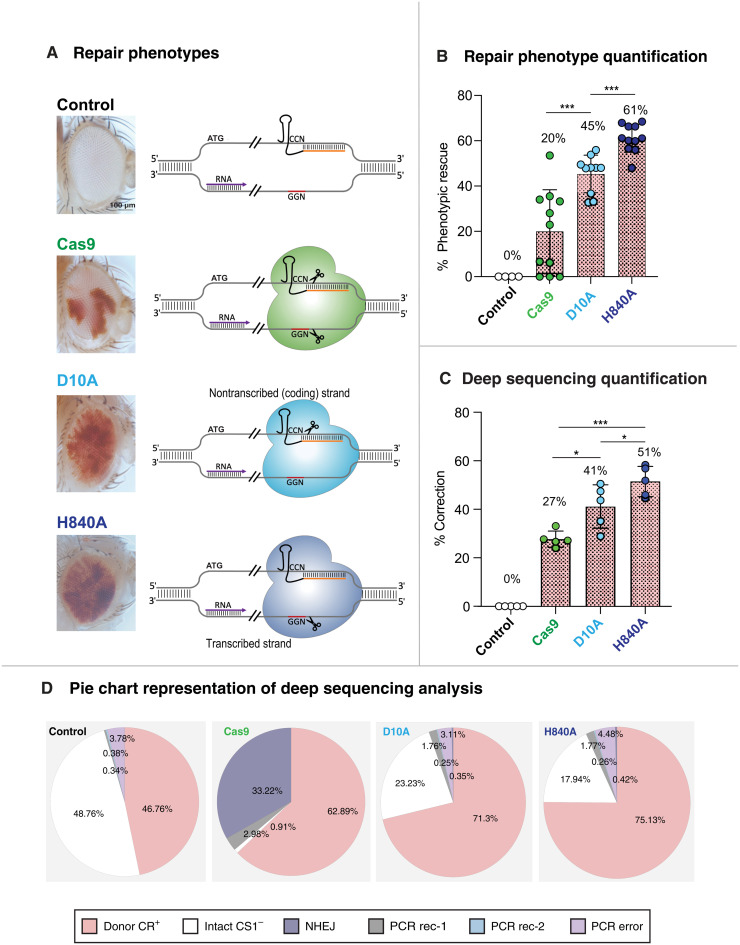
Comparison of Cas9-, D10A-, and H840A-induced repair phenotypes. (**A**) Cas9, D10A, and H840A nucleases expressed under the control of vasa promoter from three equivalent insertions (in X chromosome locus *yellow*) were used to induce allelic repair in *y*^ccw^ *w*^ATG− CR+^/*y*^vasaCas9^ *w*^ATG+ CS1−^, *y*^ccw^ *w*^ATG− CR+^/*y*^vasaD10A^ *w*^ATG+ CS1−^, and *y*^ccw^ *w*^ATG− CR+^/*y*^vasaH840A^ *w*^ATG+ CS1−^ females. Control animals were *y*^ccw^ *w*^ATG− CR+^/*y*^+^ *w*^ATG+ CS1^. Left panels show typical repair phenotypes, and diagrams on the right represent cutting or nicking by each different nuclease. (**B**) Allelic repair was quantified by image analysis using ImageJ. Nickases are more efficient than Cas9 in eliciting HTR, and H840A is more efficient than D10A for repairing the CS1^−^ allele. (**C**) Quantitative analysis of HTR of the CS1^−^ allele by deep sequencing. Correction percentages after adjustments (see Materials and Methods) from five independent reads were plotted for each genotype (no nuclease control CR^+^/CS1^−^, +Cas9, +D10A, and +H840A). Results confirm the trend calculated using pigment and Sanger sequencing analysis: Repair by D10A is significantly higher (41%) than by Cas9 (27%). H840-elicited repair (51%) is significantly more efficient than by D10A. ***P < 0.001 and *P < 0.05. (**D**) Pie chart representation of deep sequencing analysis. Color coding—pink, donor CR^+^ alleles; white, intact CS1^−^ alleles; dark purple, NHEJ mutations (centered at cut site); gray, PCR-induced recombination #1 and some asymmetrical HTR and NHEJ events (only for Cas9, D10A, and H840 samples; see fig. S9); light blue sectors, PCR-induced recombination #2; light purple sectors, PCR-induced substitutions. This representation allows global visualization of different categories of events following Cas9-, D10A-, and H840-dependent cleavage in *w*^ATG− CR+^/*y*^+^ *w*^ATG+ CS1−^ individuals. Nickases are more effective at producing HTR than Cas9, with H840 inducing the highest levels of conversion. Cas9 induces high levels of NHEJ events, while D10A and H840 only elicit low levels of NHEJ and leave ~18 to 23% of intact CS1^−^ alleles.

We took our analysis further by comparing the relative efficiencies of HTR processes following DSB or nicks targeting opposing DNA strands. For these experiments, we made use of three equivalent transgenic cassettes inserted at the same site to express either Cas9, D10A, or the alternate H840A nickase at identical levels to repair the CS1^−^ allele (*23*). H840A is mutated in the HNH catalytic domain and thus produces SSB on the opposite nontargeted DNA strand from that cleaved by D10A. Pigmentation phenotypes revealed that H840A also produced HTR phenotypes similar in pattern to those of D10A. H840A, however, was consistently more efficient than D10A in restoring *w^+^* gene function of the CS1^−^ allele (61 and 45%, respectively), while Cas9 again produced lower percentages of correction (20%; Fig. 3, A and B). Consistent with this phenotypic assessment, deep sequencing analysis revealed that the H840A nickase, which cleaves the transcribed strand in our system (Fig. 3A), led to significantly higher HDR levels than the D10A variant cleaving the opposite nontranscribed strand (51% for H840A and 41% for D10A; Fig. 3C). As observed previously, Cas9 cleavage generated a large proportion of NHEJ mutagenic events (~33%) in addition to HDR-mediated allelic repair for the CS1^−^ allele (~27%; Fig. 3, C and D). Most of these alleles consisted of deletions ranging from 1 to 83 nt, which were located primarily on the 3′ side of the gRNA cleavage site. The large size of the predominant deletions suggests that they may have been produced through microhomology-dependent mechanism by theta-mediated end joining (*24*). As expected, Nickases induced very few NHEJ mutations (~0.4%), and the few recovered events consisted predominantly of deletions 3′ to the cut site when elicited by D10A and, more frequently, 5′ to the cut site in response to H840A-dependent nicking (fig. S9). For both Nickases, a large fraction (~20%) of CS alleles remained intact, in contrast to Cas9, which converted or mutated nearly all CS1^−^ alleles, leaving fewer than 1% of them unaltered (Fig. 3D).

### Nickase-induced HTR is elevated in tissues with higher transcriptional activity

Experiments described above analyze sequences obtained from whole adult animals. We explored potential differences in repair between various organs by comparing efficiency of the HTR process evaluated by Sanger sequencing in different organs and tissues of animals expressing Cas9, D10A, or controls (fig. S10). We found that for D10A, the highest levels of HTR were found in the gut and Malpighian tubules (~70%), tissues where the *w* gene is highly expressed from the third larval instar period throughout adulthood [(*25*, *26*) and https://flybase.org/cgi-bin/rnaseqmapper.pl?dataset=tissues_stranded&xfield1=FBgn0003996]. In contrast, no increased repair was observed in heads, eyes, and carcasses, in which *w* is expressed at lower levels. Inversely, Cas9-induced HTR was not increased in guts and Malpighian tubules (fig. S10). Overall, these correlative observations suggest that transcription may contribute positively to repair outcomes induced by SSB but not DSB. Since repair is nonetheless observed even in tissues with lower expression levels of *w*, there does not appear to be an absolute requirement for high-level transcription to sustain robust HTR, suggesting that other factors also influence efficiency this process (fig. S10).

### Nickase also sustains cassette copying, albeit less efficiently than Cas9

We reported recently that transgenic cassettes referred to as “CopyCatchers” can be successfully copied to a naïve homologous site in *Drosophila* somatic cells upon targeted DSB at their site of insertion. These elements, placed into an intron, create a loss-of-function (l-o-f) allele by inserting a fluorescent reporter in-frame with the targeted endogenous gene. When combined in cis with an ATG^−^ point mutation, CopyCatchers reveal HDR events by generating mutant phenotypes coinciding with DsRed fluorescent marker expression (*18*). For example, in *w*^ATG− [cc]^/*w^+^* females expressing Cas9, DNA cleavage targeting the intact *w*^+^ allele during development results in *w*^−^ clonal phenotypes, in which somatic gene conversion leads to production of l-o-f homozygous *w*^[cc]^/*w*^[cc]^ clones also expressing the DsRed reporter (fig. S11). In *w*^ATG− [cc]^/*w^+^* females expressing D10A, we consistently observed few small *w*^−^ eye patches also expressing DsRed (fig. S11). We conclude that the D10A nickase can also mediate somatic copying of a gene cassette but does so less efficiently (~5- to 6-fold reduction) than Cas9 in this particular configuration, a possible consequence of local misalignment imposed by the CopyCatcher insertion.

In aggregate, our observations reveal that both Cas9 and Nickases lead to interhomolog allelic correction in somatic cells but that they display significantly different dynamics and efficiencies (Table 1). HTR is more efficient for repair of allelic variants in response to Nickase-induced SSB than to Cas9-dependent DSB. In addition, Nickases act later in development and do not generate mutations in contrast to Cas9, for which HTR competes with the NHEJ repair pathway.

### Cas9- and Nickase-mediated allelic correction in symmetric genetic configurations

**Fig. 4.**
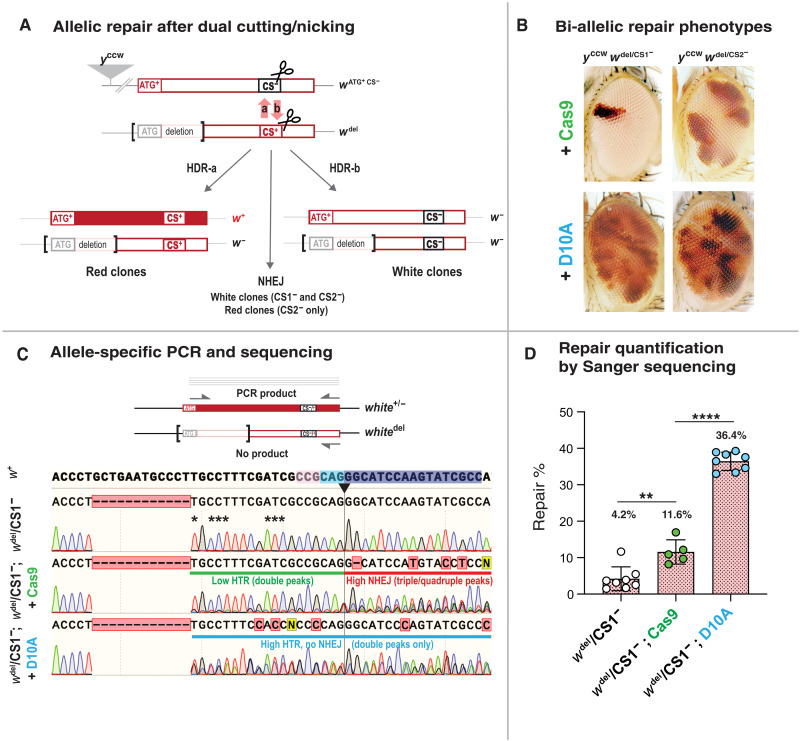
Particular configuration for allelic repair: bi-allelic HTR. (**A**) Allelic repair involving two cut-sensitive (CS) alleles: ATG^+^ CS^−^ and *w*^del^ CS^+^. Cleavage of the CS^−^ allele and repair using homologous CS^+^ sequences (HDR-a) leads to functional ATG^+^ CS^+^ combinations. Cleavage of CS^+^ and repair using CS^−^ (HDR-b) leads to a nonfunctional combination (*w*^del^ CS^−^). In both cases, the repaired allele remains cut sensitive and susceptible to additional nuclease attacks, leading to functional or nonfunctional NHEJ alleles (Cas9) or mostly remaining intact (D10A). (**B**) Clonal *w*^+^ phenotypes reveal repair of CS1^−^ (left images) and CS2^−^ (right images) alleles by Cas9 (top images) or D10A (bottom images). When both alleles are cut sensitive (CS1^−^/CS^+^), Cas9-dependent cleavage elicits very little repair (top left image). In contrast, CS2^−^/CS^+^ flies expressing Cas9 display greater repair, indicating prominent NHEJ frame-restorative repair of CS2^−^. For D10A-expresssing animals, both CS1^−^/CS^+^ (left bottom image) and CS2^−^/CS^+^ (right bottom image) display similar repair phenotypes consisting of numerous small *w*^+^ clones, reflective of predominant HTR. (**C**) Allele-specific sequencing reveals HTR in CS1^−^/CS^+^ genotypes (top diagram). The forward primer located at the initiation codon ensures selective amplification of the ATG^+^ allele. In control animals (no nuclease, top chromatogram), only CS1^−^ sequences are amplified. Asterisks indicate peaks used for quantitative analysis in (D). Following Cas9-induced DSB (second electropherogram), double peaks starting at the CS1^−^ deletion break point (green line) reveal effective HTR. Triple and quadruple peaks starting at the cut site reveal high-level NHEJ. Following D10A-induced nicks (third electropherogram), more asymmetrical double peaks reveal higher levels of HTR (blue line), while no NHEJ is detectable after the cut site. (**D**) Quantitative analysis of HTR by allele-specific sequencing in CS1^−^/CS^+^ animals. Correction percentages are plotted for each genotype. Cas9-induced repair (~12%) is significantly lower than D10A-induced repair (~36%). ****P < 0.0001 and **P < 0.01.

Experiments described above involve allele-specific DNA cleavage, after which cut-resistant sequences are used for directional repair at the homologous site. We tested an alternate “sensitive/sensitive” configuration, for which both alleles of the *w* locus are subjected to DNA cleavage. For these experiments, we made use of the *w*^1118^ null allele (referred to as *w*^del^ hereafter), a multikilobase deletion encompassing the first exon and most of the first intron of *w* (*27*), yet leaving the *white*-gRNA recognition site and adjacent sequences intact (Fig. 4A). Trans-heterozygous *w*^del^/*y*^ccw^ w^ATG+ CS1−^ flies produced only rare small red clones in response to Cas9 expression (Fig. 4B, top left), indicating that bi-allelic DSB rarely resolves in successful restoration of functional *w*^+^ alleles. In contrast, *y*^ccw^ *w*^ATG+ CS1−^/*w*^del^ females expressing D10A displayed frequent *w*^+^ clones (Fig. 4B, bottom left), albeit fewer than observed in comparable flies carrying a cut-resistant CR^+^ allele (Fig. 2A). These results suggest that each allele (*w*^CS1−^ and *w*^del^) can be cut and repaired using the other allele as a template. While the *w*^ATG+ CS1−^ allele may be repaired using wild-type homologous sequences, leading to a functional *w*^+^ allele, the *w*^del^ allele repaired using CS1^−^ sequences always remains nonfunctional. In either case, both alleles remain sensitive to further cleavage, however additional copying events typically will no longer alter the nature of the repaired allele (functional versus nonfunctional; Fig. 4, A and B). In the presence of Cas9, this repeated assault may lead to the ultimate accumulation of NHEJ mutations and a permanent loss of gene function in most cells. The presence of numerous *w*^+^ clones in *y*^cc^ *w*^ATG+ CS2−^/ *w*^del^; Cas9 animals (in which the CS2^−^ allele can be restored to a functional state through NHEJ), but not in equivalent CS1^−^ flies, strongly supports this hypothesis (Fig. 4B).

We further characterized HTR events following bi-allelic cleavage/nicking by performing sequence analysis on control and gene-edited flies. We detected and quantified HTR events by performing selective polymerase chain reaction (PCR) amplification of the *w*^CS1−^ allele using a primer annealing to the ATG initiation codon region (which cannot amplify sequences from the *w*^del^ allele since it lacks the ATG initiation codon and surrounding sequences) for this selective amplification (Fig. 4C). Sequence analysis of such PCR products revealed only the expected CS1^−^ 12-nt deletion in control *w*^del^/*w*^ATG+ CS1−^ flies (Fig. 4C). In contrast, reads from similar flies expressing D10A displayed double peaks corresponding to overlapping wild-type and CS1^−^ alleles and revealing frequent instances of allelic repair (Fig. 4C, bottom). Quantification of such Sanger sequencing data revealed a repair rate of ~36% (Fig. 4D), which, as expected, is lower than the 52% correction estimate in previous experiments involving directional repair using a cut-resistant donor allele and the same D10A source (Fig. 2D). In contrast, *w*^del^/*w*^ATG+ CS1−^; Cas9 animals exhibited only low levels of correction (~12%; Fig. 4D) and high levels of NHEJ-induced mutations visible as triple and quadruple peaks encompassing the cut site (Fig. 4C, middle row). These observations are consistent with our proposed mechanism for the sensitive/sensitive configuration: Each allele can be replaced by the other, resulting in a homozygous state for either allele (CS1^−^ or CS^+^). Because these alleles are both cut-sensitive, they remain the target for further cleavage/nicking, unless NHEJ produces cut-resistant (and likely) nonfunctional alleles. This latter outcome is frequent with Cas9 but rare for D10A, as supported by both eye phenotypes and sequence analysis (Fig. 4, A and D).

### Allelic correction does not require long-range stable chromosome pairing

All repair processes examined above involve copying from an allele present on the homologous chromosome. These events are likely to be facilitated by long-range chromosomal pairing, a phenomenon that is central to crossing over in the germ line of multicellular organisms but that is also prevalent in somatic tissues of dipterans underlying phenomena such as transvection (*28*–*30*). Interhomolog pairing, while thought to be rare in typical mammalian cells, has been reported in some cancerous cell lines and can be induced locally by DSBs (*17*, *31*–*33*).

**Fig. 5.**
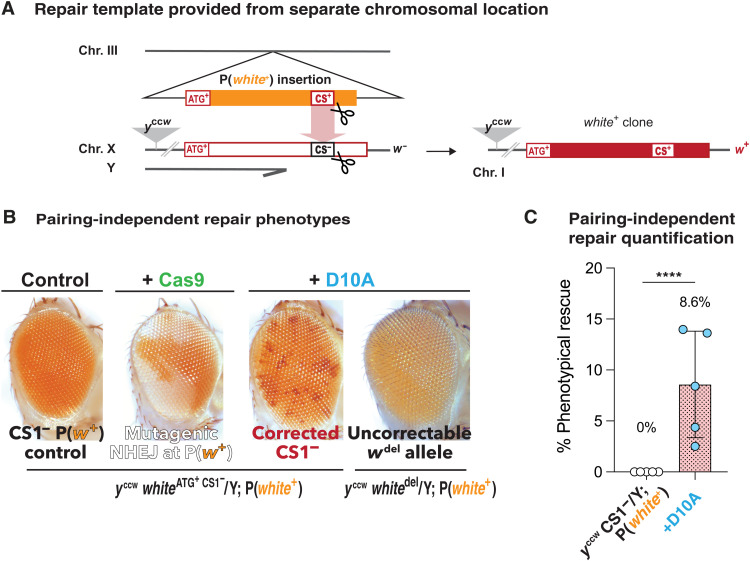
Evidence for pairing-independent allelic repair. (**A**) System designed to reveal pairing-independent HDR by *white*^+^ clonal phenotypes, where repair template (transgene carrying a *mini*-*white* cDNA) is provided from a separate chromosomal location, after D10A-induced nicking. (**B**) An autosomal P(mini-*white*^+^) transgenic insertion causes a light orange eye phenotype in otherwise *w*- background (left bottom). In y^ccw^ w^ATG+ CS1−^/Y; P(mini-*white*^+^)/vasaCas9 males (second), efficient DNA cleavage at the P(mini-*white*^+^) insertion causes large *white*^−^ clones derived from NHEJ-mediated mutagenesis. In y^ccw^ w^ATG+ CS1−^/Y; D10A/+; P(mini-*white*^+^)/+ males (third), numerous small *white*^+^ clones in orange background indicate that allelic repair is occurring consistently using sequence homology, independently of chromosome pairing. In y^ccw^ *w*^del CS+^/Y; D10A/+; P(mini-*white*^+^)/+ males (fourth), such repair is not detectable, as the *w*^del^ cannot be restored to a functional state. This latter control demonstrates that the *white*^+^ clones in y^ccw^ *w*^ATG+ CS1−^/Y; D10A/+; P(mini-*white*^+^)/+ animals (third) do not result from any alteration of the P(mini-*white*^+^) sequences but repair of the CS1^−^ allele from the autosomal the P(mini-*white*^+^) sequences. (**C**) Quantification of pairing-independent repair of the CS1^−^ allele reveals ~8% phenotypic rescue induced by D10A compared to no rescue in control. In these experiments, the second chromosome vasaD10A and the third chromosome vasaCas9 (as in Figs. 1 and 2) were used. ****P < 0.0001.

We tested whether allelic repair might also occur in the absence of somatic chromosomal pairing in our *Drosophila* system using available transgenes carrying a mini-*white* cDNA that result in variable eye pigmentation phenotypes when inserted at different chromosomal locations. Insertions were selected for the light eye color they produced (when placed in a *w*^−/−^ background), so that repair events could be distinguished as dark red clones contrasting with yellow or orange background. *y*^ccw^ *w*^ATG+ CS1−^/Y individuals carrying a P<mini-*white*^**+**^> insertion (Fig. 5A) were examined for clonal phenotypes elicited by SSB (D10A) or DSB (Cas9). In the absence of any nuclease, eyes appeared uniformly orange, resulting from expression of the intact mini-*white*^**+**^ P-element marker gene. Individuals carrying a source of Cas9 displayed *w*^−^ clones that covered a substantial fraction of the eyes (Fig. 5B, left middle), presumably reflecting the P<mini-*white*^+^> sequences having been targeted for DSB and repaired through the error-prone NHEJ pathway. When the D10A nickase was assayed in the same genetic context, we instead recovered many small red clones across the eye surface (Fig. 5B, middle right). D10A-induced repair frequencies ranged from ~2 to 14% as evaluated by image-based quantification (Fig. 5C and fig. S12). Because we used the in-frame deletion CS1^−^ allele for these experiments, the observed functional repair can only be attributed to HDR and not to an alternative mutagenic process affecting the P<mini-*white*^**+**^> insertion. When the *w*^[del]^ allele (which cannot be restored to a functional state by allelic correction) was used instead of the CS1^−^ allele (in *y*^ccw^ *w*^[del]^/Y; D10A/; P<*mini*-*white*^**+**^>/+ individuals), no red clones were generated (Fig. 5B, right most).

We also tested two other autosomal P<*mini*-*white*^+^> insertions for their ability to serve as templates for such pairing-independent correction. We found similar small red clone phenotypes with varying frequencies induced by D10A but not Cas9 (fig. S12), indicating that this form of homolog-independent repair does not strictly depend on a particular genomic location of the repair template. We conclude that allelic correction can occur in a fashion relying only on sequence homology within the *mini-white* gene but not on long-range chromosome pairing, a process we refer to as pairing-independent repair. While similar pairing-independent repair has been demonstrated in the *Drosophila* germ line, induced by Cas9 (*34*) or by the P-element transposase (*35*), the present experiments reveal that this process can also take place in somatic tissues. As in these previous studies, different chromosomal positions and expression levels of the donor insertions are likely to greatly influence the efficiency of the pairing-independent repair.

Cumulatively, these varied genetic and molecular assessments demonstrate that highly efficient somatic allelic conversion at the *white* locus operates after allele-specific or bi-allelic targeted cleavage or nicking. This process is promoted more efficiently by nonmutagenic D10A or H840A nickases than by Cas9 and does not depend strictly on chromosome pairing, although such pairing increases correction frequencies (summarized in Table 1).

## DISCUSSION

In this study, we developed a versatile genetic system in which targeted DNA breaks created either by Cas9 or Nickases elicit distinct repair processes revealed by quantifiable pigmentation phenotypes in *Drosophila*. We used a variety of allelic combinations and showed that HDR processes using cut-resistant sequences from the homologous chromosome as repair templates (HTR) are unexpectedly efficient in somatic cells. While interhomolog repair following DSB has been observed in the *Drosophila* germ line (*36*–*38*), it has been only recently reported (*18*, *19*) and is much-less characterized in somatic cells where the NHEJ pathway is thought to prevail and operates throughout the cell cycle to repair DNA breaks (*8*).

The most notable finding of our study is that two Cas9-derived mutant nucleases, D10A and H840A, which nick rather than cleave target DNA, sustain high rates of somatic allelic conversion (45 to 65%) that exceed those observed with Cas9 (~30%). Patterns of such Nickase-induced SSB repair appear distinct from those resulting from Cas9-induced DSB repair. Nickases produced far fewer NHEJ mutations and initiated HTR at later phases of development than Cas9, as revealed by the smaller sizes and higher numbers of clones. D10A can sustain somatic copying of multikilobase insertions but does so much less efficiently than Cas9 (the reverse of their activities for allelic repair; fig. S11). In addition, D10A supports only low-level germline copying of cleavage-resistant alleles, while Cas9 does so with great efficiency (summarized in Table 1).

These notable differences in Nickase versus Cas9 activity can be interpreted considering previous knowledge of DSB and SSB repair mechanisms. DSBs are repaired either through mutagenic NHEJ or HDR, which involves copying from the sister chromatid (during S and G_2_ phases) or from the homologous chromosome (during all phases or cell cycle). Both processes will give rise to noncleavable sequences (NHEJ-induced mutations or copying the cut-resistant allele from the homologous chromosome), such that an end point is reached early after the first few repair cycles, generating large solid *w*^+^ and *w*^−^ clones in our system. In contrast, DNA nicks are generally repaired precisely through direct religation (*39*, *40*), thereby restoring an intact cut site, which is then amenable to iterative nicking. Thus, repeated nick-and-repair cycles are likely to take place until conditions favoring or requiring HDR arise, wherein copying of cut-resistant sequences from the homologous chromosome terminates this cyclic process.

Two potential processes likely to promote nick-induced HDR are transcription and replication, both of which may favor the occurrence of a second nick on the opposing strand. In this instance, juxtaposition of two SSB on opposing strands may result in effective DSB with overhangs of varying sizes (*39*, *40*), which may then be amenable to repair by standard or alternate HDR processes. We observed a correlation between D10A-induced (but not Cas9-induced) HTR and elevated *white* expression in the digestive and secretory systems (fig. S10), supporting models in which transcription favors such SSB to DSB transition (*41*, *42*). During replication, nicks created by topoisomerases required to relieve strain produced by supercoiling (*43*) may also resolve into DSB when generated in proximity to nickase-induced SSBs. Alternatively, proximity of junctions between Okazaki fragments or physical strains exerted on single-strand intervals may also result in secondary breakage events. Unlike Cas9-induced DSBs, such DSBs generated indirectly by Nickase are likely to occur at a much slower pace, potentially explaining the delayed developmental timing of Nickase-induced repair compared to Cas9-induced repair. This contrasting dynamics could also explain the far lower rate of NHEJ mutagenic events induced by Nickases (~0.4%) compared to that of Cas9 (~30%), as the choice between the NHEJ (faster repair) and HDR (slower repair) is likely to be influenced by the rate of DSB production.

Previous mechanistic and genetic studies suggest that HDR induced by nicks acts either through the canonical Rad51 and BRCA2 factors or through an efficient alternative Rad51-independent pathway (*44*). In this study, nicking the transcribed DNA strand led to a higher level of repair than nicking the coding strand, as observed also in our system (Fig. 3), while producing two nicks on opposite DNA strand produces efficient HDR (*45*). In addition, increased levels of the RecQ5A helicase have been shown to favor a shift toward this alternate pathway (*46*). In *Schizosaccharomyces pombe*, nick-induced HDR using the sister chromatid as repair template can be visualized in PNKP^−^ (polynucleotide kinase-phosphatase) mutants. The absence of PNKP activity prevents direct religation and promotes a Rad51-independent HDR pathway (*47*). Another example has been documented in birds in which pseudogene-templated gene conversion is essential for generating immunoglobulin diversity and is initiated by DNA nicks at the V segment of the light chain locus (*48*, *49*). These established examples of HDR induced at nicks in somatic eukaryotic cells, ranging from yeast to humans, suggest that these processes rely on broadly conserved repair machinery. As our allelic repair system in *Drosophila* is sensitive and quantitative, it should be amenable in future studies for screening RNA interference and misexpression lines to potentially identify conserved factors critical for Cas9- or Nickase-induced HTR.

Dipteran insect chromosomes differ from those of mammals by engaging in extensive somatic pairing (*17*, *31*, *50*) in both germline and somatic tissues, as illustrated by the phenomenon of transvection, wherein regulatory sequences from one chromosome promote expressing of coding sequences from the homologous chromosome (*28*–*30*). Thus, such somatic chromosome pairing is expected to play an essential role in favoring HTR observed in our system. However, we found that nick-induced HDR can also operate, albeit with reduced efficiency, when the homologous donor DNA is provided from a distinct chromosomal location. Similar pairing-independent gene conversion has been achieved in the insect germ line for which success depends highly on respective chromosomal position of donor and recipient sequences (*34*, *35*). In mammalian cells, only few instances of interhomolog repair have been reported. For example, Cas9-induced DNA breaks can result in directional copying of a gene cassette onto the homologous chromosome in human HEK293T (human embryonic kidney 293T) cells (*18*) and homozygosity of a cut-resistant allele in mouse embryos when Rad51 is provided in excess (*19*). Such phenomenon might be favored by local interhomolog pairing that has been observed following DBSs (*32*, *33*).

CRISPR-based gene editing offers great promise for gene therapy. However, numerous reports have raised concerns regarding Cas9-dependent production of large deletions and their potential deleterious off-target activities (*51*, *52*). As is widely documented in various vertebrate and invertebrate systems, Nickases offer the desirable feature of causing far fewer NHEJ-generated mutations and off-target effects than Cas9 (*20*, *44*, *49*, *53*). Thus, nick-induced HTR could offer a safe and potentially effective approach for such therapeutic applications. Findings summarized above suggest that interhomolog HTR at nicks may be achievable also in mammalian systems. Because vertebrate chromosome pairs are generally thought to remain separated in different chromosomal territories (*17*) as indicated by numerous studies involving DNA fluorescence in situ hybridization and Hi-C analysis (*54*–*56*), HTR in mammalian cells and tissues is likely to require future extensive optimizations guided by results obtained in *Drosophila* to achieve desirable efficiency. For example, slow continuous delivery of CRISPR components over the course of several days as is the case in our study may prove beneficial over one-time delivery approaches. In addition, the pairing-independent allelic conversion paradigm we describe here may serve as an excellent model for enhancing nick-induced allelic correction in both fly and mammalian systems. If the frequency of such events could be increased either by promoting interhomolog pairing or by optimizing nick-specific repair processes, then such strategies could be harnessed to correct numerous dominant or trans-heterozygous disease-causing mutations.

## MATERIALS AND METHODS

### Plasmid construction

To create the *white*^−^ cut-sensitive alleles and the ATG^−^ mutation described in Fig. 1 and fig. S1, three plasmids were constructed to express the 5′-gRNA, the 3′-gRNA, or the ATG-gRNA. Annealed oligos were inserted into the PCFD3 vector after digestion with Bbs1 as described on http://crisprflydesign.org/plasmids/. Sequences of the three pair of oligos were (i) 5′-gRNA, GTCGGAAAGGCAAGGGCATTCAGCA (forward) and AAACTGCTGAATGCCCTTGCCTTTC (reverse); (ii) 3′-gRNA, GTCGGCCATTGAGCAGTCGCATCC (forward) and AAACGGATGCGACTGCTCAATGGC (reverse); (iii) ATG-gRNA, GTCGAGTGTGAAAAATCCCGGCAAT (forward) and AAACATTGCCGGGATTTTTCACACT (reverse).

### Microinjection of gRNA constructs and establishment of *w*^−^ lines

Plasmids were prepared using the Qiagen Plasmid Midi kit (#12191), and sequence was checked. Each gRNA construct was coinjected with a transient source of Cas9 (pAct-Cas9, Addgene plasmid no. 62209) by Rainbow Transgenics. Injection mixes were assembled with each gRNA plasmid (final concentration: 500 ng/μl) and pAct-Cas9 (final concentration: 500 ng/μl) in a volume of 50 μl. Injection mixes for all gRNA constructs were injected into an Oregon-R (*white*^+^) stock (Bloomington Drosophila Stock Center, BDSC #2376). *White*^−^ mutant males were selected in F1 progeny to establish isogenic lines, and specific alterations were determined by sequencing.

### *Drosophila* genetics

The *y*^ccw^ insertion was described in figure S2 of (*21*). It is inserted in the *yellow* locus and carries two gRNAs: one (*yellow*-gRNA) targeting its own site of insertion (promoting initial integration and copying of the *y*^ccw^ cassette) and the other (*white*-gRNA) targeting cleavage in the third exon of the *white* gene. The *y*^ccw^ insertion identified by DsRed fluorescence in eyes was recombined with the ATG^−^ mutation associated with the CR^+^ allele using Cas9-mediated allelic conversion to generate the *y*^ccw^ ATG^−^ CR^+^ donor (cut-resistant) line (DsRed^+^ *white^−^*) used throughout this study. Cas9 was expressed from the third chromosome insertion PBac{vas-Cas9}VK00027 (BL#51324, marked with 3XP3-GFP), which expresses Cas9 in the germ line and somatically. D10A was expressed from the second chromosome insertion (*y*1 *w*^1118^ P(3xP3-EGFP, vasa-cas9D10A)attP40A, a gift from A. Rodal (Brandeis University). Lines were established combining CS^−^ alleles and each nuclease, and males from such lines were crossed to females from *y*^ccw^ ATG^−^ CR donor lines to produce experimental animals. For comparing the activity of the different nucleases and deep sequencing analysis (Fig. 3), three lines were designed in which vasaCas9, vasaD10A, or vasaH840A sequences associated with the DsRed marker were inserted at the same location in the *yellow* gene (*23*). For pairing-independent HDR (Fig. 5 and fig. S12), the following autosomal P(*white*^+^) insertions producing orange eye phenotypes were used: BL#1799 (P{GAL4-Hsp70.PB}89-2-1, chromosome 3), BL#2077 (P{GAL4-Hsp70.PB}2, chromosome 2), and BL#1822 (P{GAL4-Hsp70.PB}31-1, chromosome 3).

### Sequence analysis of allelic correction

To establish sequences and correction percentages shown in Fig. 2, genomic DNA was extracted from individual flies of relevant genotypes (*y*^ccw^ ATG^−^ CR^+^/*y*^+^ ATG^+^ CS1^−^; ± vasaCas9/vasaD10A). PCR reactions were assembled using the Q5 Hotstart master mix (New England Biolabs, #M0494S) with the following primers: CTGCTCATTGCACTTATCTACAAG (forward) and GCAAATTAAAATGTTACTCGCATCTC (reverse).

PCR products (2.2 kb) were purified before Sanger sequencing with internal primers: CS1^−^ allele, GCTGGTCAACCGGACACGCGG (forward) and CS2^−^ allele, CTCGCTGCCGATAGGTCAGATGTCG (reverse). To evaluate correction percentages, seven peaks (marked with the * symbol in Fig. 2C) located in the CS1^−^ 12-nt deletion were chosen for consistent low distortion (similar peak heights for the CS1^−^ and CR^+^ alleles in heterozygous control animals). For each peak, correction percentage was calculated using the formula: [pv(CR^+^) − pv(CS1^−^)] × 100/[pv(CR^+^) + pv(CS1^−^)], where “pv” refers to the peak value of the indicated allele (CR^+^ or CS1^−^) read in the SNAPgene program. Seven “low distortion” peaks located between the cut site and the CS2^−^ 1-nt insertion were chosen for quantifying HTR of the CS2^−^ allele.

In Fig. 3C, allele-specific PCR was performed using a forward primer specific for the wild-type ATG^+^ allele to generate a 3.7-kb product: GTGTGAAAAATCCCGGCA**ATG**G (forward) and AGGGAGCCGATAAAGAGGTCATCC (reverse).

PCR products were sequenced with the same internal primer as in Fig. 2C. Allelic correction percentages were calculated as follows:

pv(CR^+^) × 100/[pv(CR^+^) + pv(CS1^−^)], which takes in account the fact that only the receiver allele (ATG^+^) is read in these samples.

### Image acquisition

Bright-field, GFP, and DsRed eye images were acquired on a ZEISS Axio Zoom.V16 microscope at ×112 magnification with an Axiocam 506 color camera, using the Zen pro 2012 software. For each eye, *z*-stacks of 14 to 20 images at ~10-μm intervals at 20-ms exposure for the GFP channel and 50 ms for the DsRed channel were acquired. Focus stacking was performed using Helicon Focus 7.5.4 software and saved in tif format.

### Image-based quantifications

The tif images were opened in ImageJ, and brightness and contrast were adjusted using the “auto” tool (Image>adjust>bright/cont>auto). Images were converted to black and white using the Type function (8 bit) in the image menu. Using the freehand tool, the total area of the eye was encircled. Black clones were identified using the threshold function (Image>adjust>threshold). The total area of each pigmented clones was evaluated using the “Analyze particles” function (Analyze> analyze particles), while the total area of the eye was calculated using the “area” function (Analyze>set measurements>Area). The percentage of total area of all particles (areas representing repair) relative to the total area of the eye was calculated for each eye and plotted in Prism 9.2.0.

### Amplicon-based deep sequencing

Genomic DNA was extracted from group of 10 flies or single flies of each genotype. Sequences around the gRNA cleavage site were amplified by PCR using primers specifically designed for deep sequencing, with 5′ tails complementary to the Illumina partial adaptors (5′-ACACTCTTTCCCTACACGACGCTCTTCCGATCT-3′ for forward primers and 5′-GACTGGAGTTCAGACGTGTGCTCTTCCGATCT-3′ for reverse primers). Two sets of primers were designed to avoid primer-specific artifacts: set1, ACACTCTTTCCCTACACGACGCTCTTCCGATCTccaatttgaaactcagtttgc and GACTGGAGTTCAGACGTGTGCTCTTCCGATCTgtcatcctgctggacatag; set 2, ACACTCTTTCCCTACACGACGCTCTTCCGATCTgcgcccaggaaacatttgctcaag and GACTGGAGTTCAGACGTGTGCTCTTCCGATCTcgctgccgataggtcagatgtcg.

PCR products were gel-purified, and 20 ng/μl of each sample was sent for Illumina paired-end 150- to 500-bp Amplicon-based deep sequencing. All reads were analyzed using manual CRISPResso2 command lines (https://crispresso.pinellolab.partners.org/submission), with wild-type *white* gene used as reference.

To establish the percentage of allelic correction, we first established a normalization factor (NF) from the control group, which takes in account the rate of PCR errors found in each experiment. NF = 100/(%CR + %CS1 + %PCR-rec1 + %PCR-rec2). This factor brings the total percentage of events to 100% and eliminates the contribution of PCR errors. The percentage repair is then calculated as: NF × (%CRexp. − %CRcont.) × 2.

### Statistical analysis

All the experimental data presented in this study are from at least three independent experiments. Statistical data were analyzed and plotted using GraphPad Prism 9.2.0 by two-tailed *t* test. The SD is represented by error bars in bar graphs centered around the mean, and to confirm significance, *P* values were calculated and represented as follows: ****P < 0.0001, ***P < 0.001, **P < 0.01, and *P < 0.05.

## Notes

View/request a protocol for this paper from *Bio-protocol*.
