# Supplementary material for: Cas9/Nickase-induced allelic conversion by homologous chromosome-templated repair in Drosophila somatic cells
Source: Sci Adv. 2022 Jul 1;8(26):eabo0721. doi: 10.1126/sciadv.abo0721 (PMC10883370; doi:10.1126/sciadv.abo0721)
Supplement: Supplementary file 1 — Figs. S1 to S12 [file sciadv.abo0721_sm.pdf]

Science Advances
AAAS

Supplementary Materials for

Cas9/Nickase-induced allelic conversion by homologous chromosome-templated repair in *Drosophila* somatic cells

Sitara Roy *et al.*

Corresponding author: Annabel Guichard, aguichard@ucsd.edu; Ethan Bier, ebier@ucsd.edu

Sci. Adv. **8**, eabo0721 (2022)
DOI: 10.1126/sciadv.abo0721

This PDF file includes:

Figs. S1 to S12

Supplementary Figure 1

***white*⁻ mutations generated in proximity to the *white*-gRNA cut site**

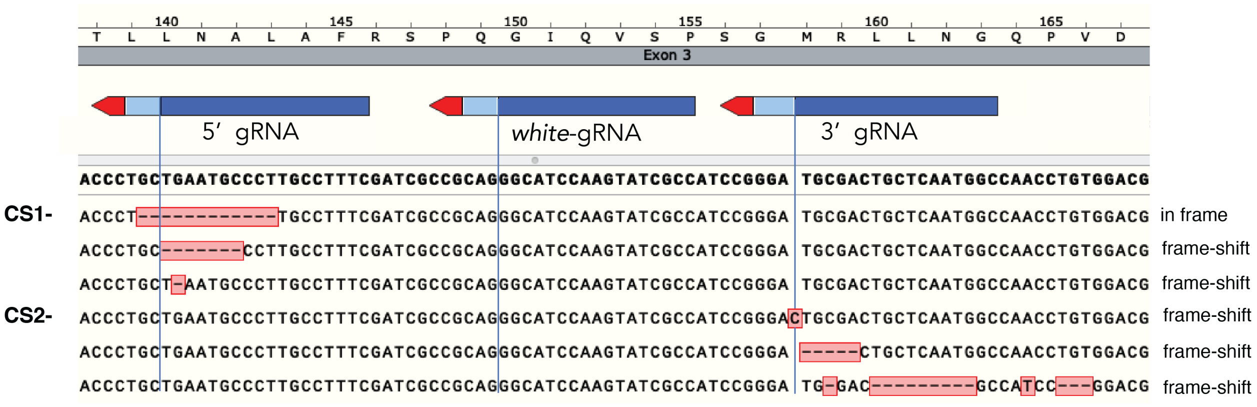

Fig. S1. Sequence of *white* ⁻ mutations generated in proximity to the *white*-gRNA cut site. Sequence of *white*⁻ mutations generated in proximity to the *white*-gRNA cut site. Top row: reference sequence with gRNA sequences highlighted in blue and corresponding PAM site in red. 5' gRNA and 3' gRNA targeting sites in proximity to the *white*-gRNA cut site were used to create *white*⁻ mutations with sequences shown below. These alleles remain sensitive to DNA cleavage directed by the *white*-gRNA (Cut-Sensitive = CS). The CS1- mutation located 5' to the y^{ccw} cut site, and CS2- mutation located on the 3' side used throughout this study are indicated. CS1- is the only in-frame mutation, while all other mutations are frame-shifts.

Supplementary Figure 2

Generation of *white*⁻ and *white*⁺ cut-resistant (CR) alleles

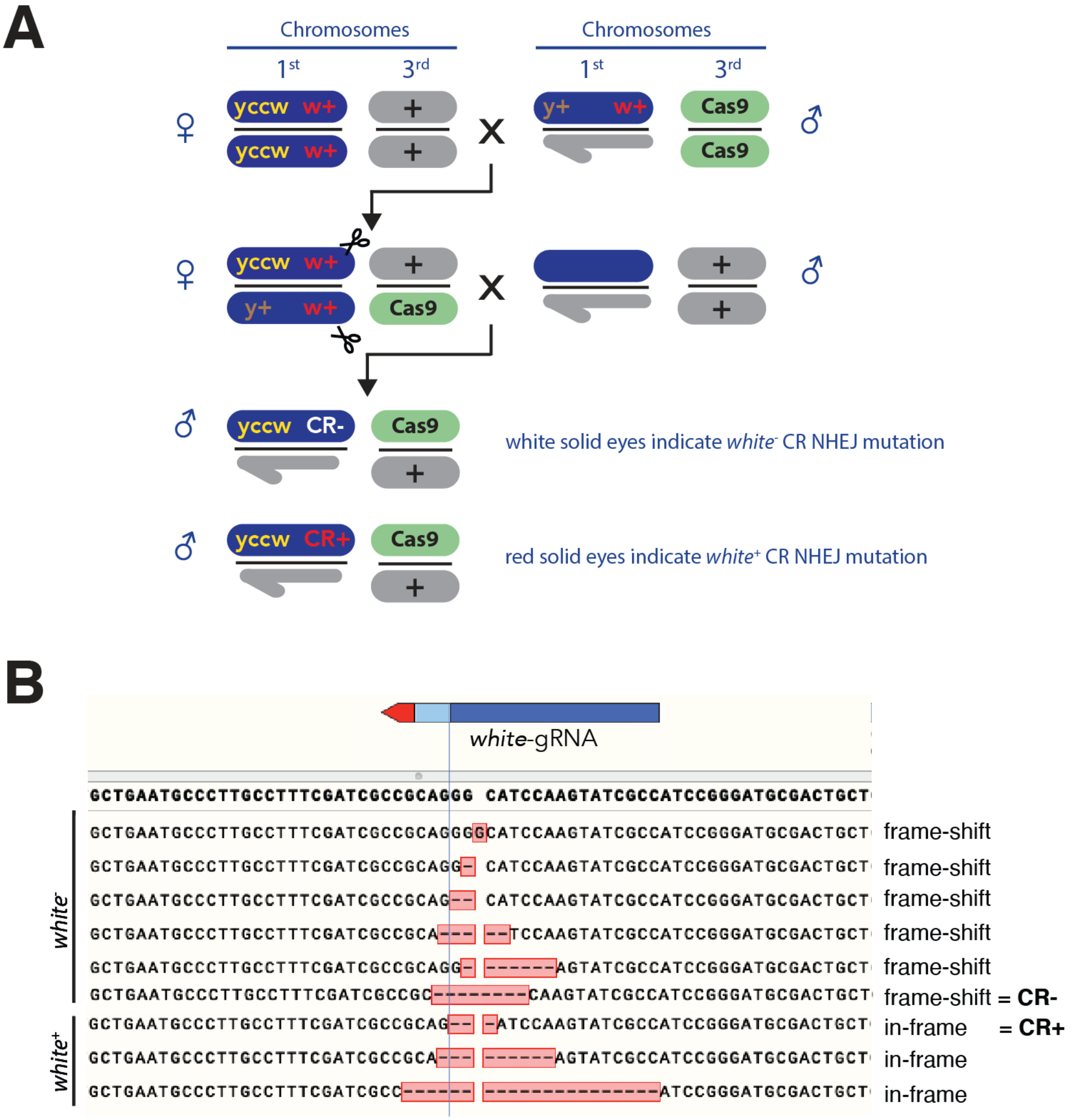

Fig. S2. Generation of *white*- and *white*+ cut-resistant (CR) alleles. (A) Cross-scheme used to generate cut-resistant CR- and CR+ alleles. F1 female carrying the y^{ccw} insertion on the X and a vasaCas9 insertion on the 3rd chromosome were crossed to wild-type CantonS (*white*$^{+}$) males to produce bi-allelic cleavage and resulting NHEJ mutations recovered in F2, as males with solid

white (w^-) eyes or red (w^+) eyes. **(B)** Sequence analysis on the lower panel shows that these F2 males carry mutations affecting the *white*-gRNA cut site, that are predicted to be cut-resistant. The first 5 sequences derive from *white*- mutants and show frame-shift alterations, while the last 3 sequences are from *white*+ mutants, and show in-frame alterations. The CR+ ad CR- alleles used in Fig. 1 and Fig. 2 are indicated.

Supplementary Figure 3

semi-quantitative grading of repair phenotypes

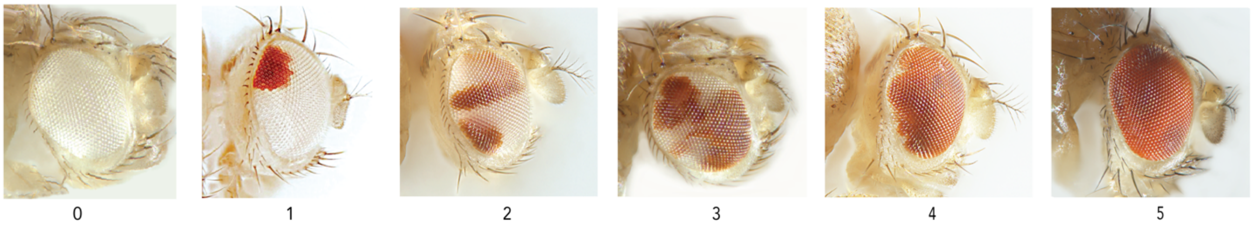

Range of Cas9-induced *white*$^{+}$ clonal phenotypes in y^{ccw} $w^{ATG- CR+}$/ $w^{ATG+ CS-}$; Cas9 /+ animals

Fig. S3. Semi-quantitative grading of repair phenotypes. Each panel shows a clonal pigmentation phenotype in y^{ccw} $w^{ATG- CR+}$ / y^{+} $w^{ATG+ CS-}$; vasaCas9 flies, with a scale ranging from 0 (no w^{+} clones) to 5 (complete w^{+} eye), used to evaluate the amount of repair in individual eyes.

Supplementary Figure 4

Allelic conversion occurs at high rates in the female germline

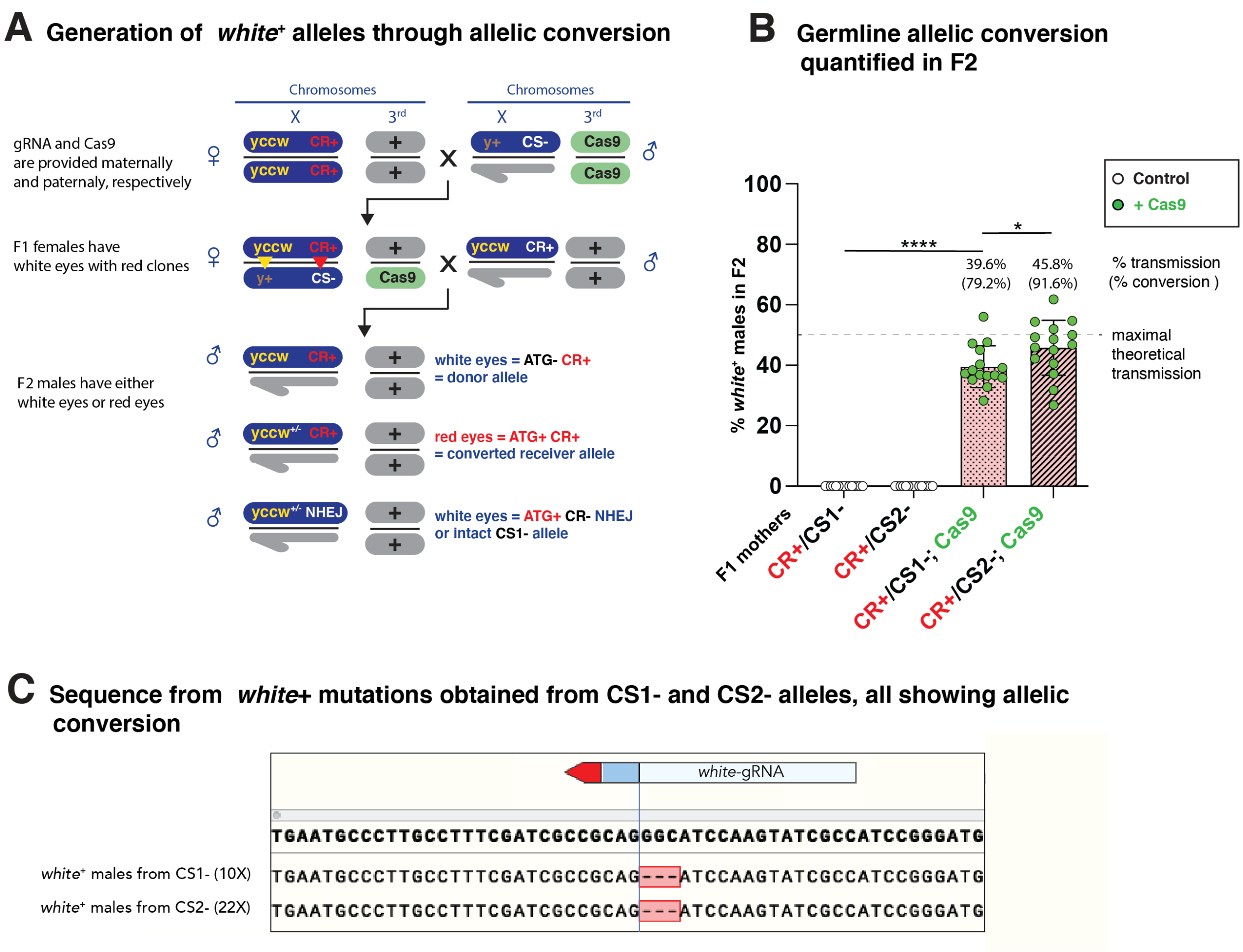

Fig. S4. Allelic conversion occurs at high rates in the female germline. (A) Cross scheme employed to recover *white*$^+$ alleles in F2 through allelic conversion. F1 y^{ccw} $w^{ATG-\ CR+}$ / $w^{ATG+\ CS-}$; vasaCas9 females produce F2 male progeny with either *w*- (donor ATG- CR+, intact CS- or unfunctional NHEJ alleles) or *w*+ eyes, which received converted ATG+ CR+ *white*$^+$ alleles. **(B)** Allelic conversion percentages for CS1- and CS2- alleles in absence (white dots) or presence of the Cas9 source (green dots). No conversion is observed in absence of Cas9, while introduction of Cas9 leads to a high rate of allelic conversion close to the maximal 50% ratio. Generation of w^+ alleles is slightly less efficient for the CS1- than for the CS2- allele. **(C)** Sequences from w^+ individual F2 males obtained from CS1- and CS2- alleles, all showing the CR+ donor allele, demonstrating that CS1- and CS2- alleles were converted by germline HTR to generate functional ATG+ CR+ alleles.

Supplementary Figure 5

Mutant alleles produced by NHEJ in male germline

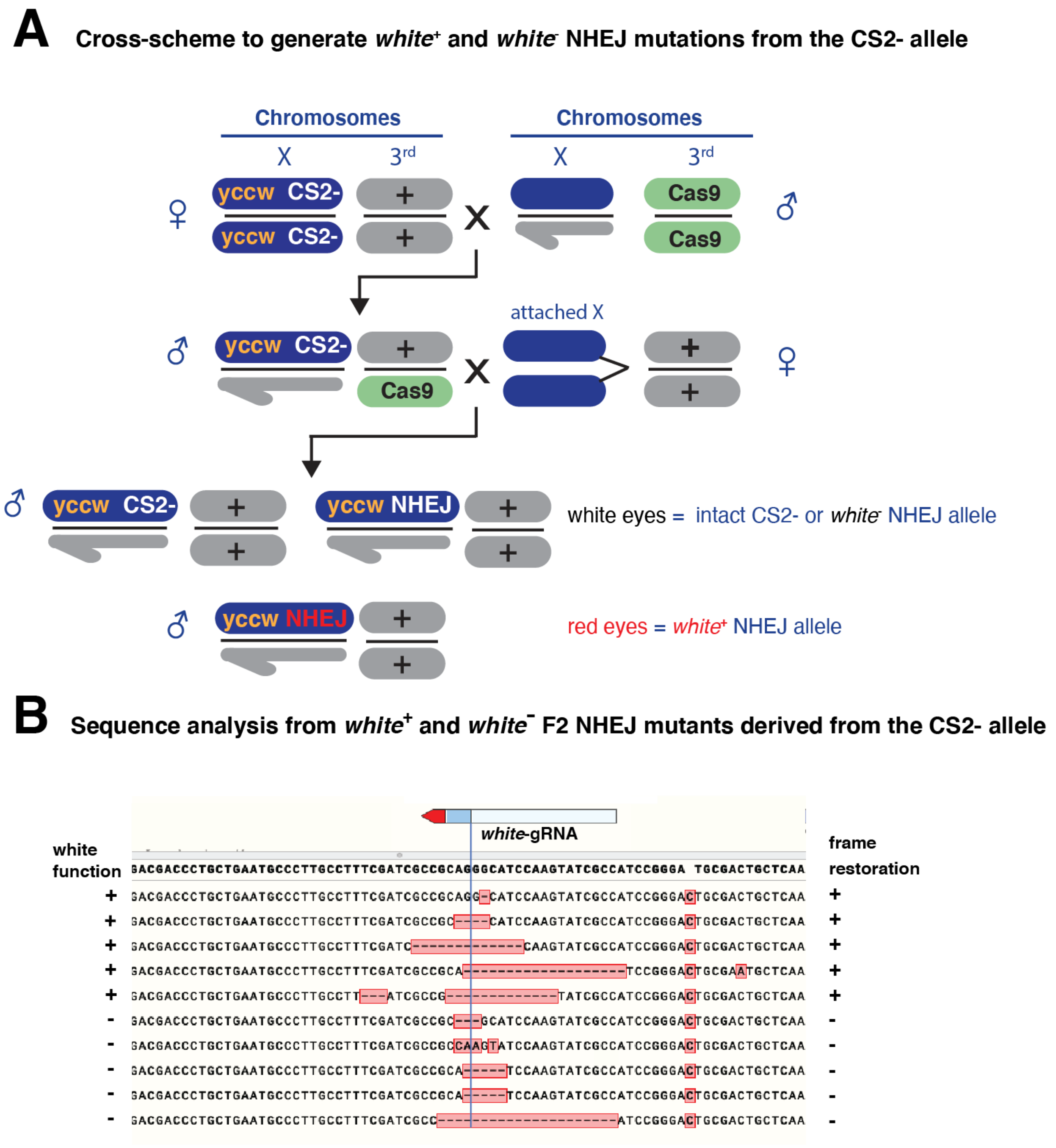

Fig. S5. Generation of F2 mutant alleles by NHEJ. (A) Cross-scheme to generate w^{+} NHEJ mutation from the CS2- allele. F1 y^{ccw} $w^{ATG+\ CS2-}$/Y ; vasaCas9/+ males are crossed to attached-X females to produce F2 males inheriting the X chromosome paternally. w^{-} and w^{+} NHEJ mutants

were selected for sequencing. **(B)** Sequence analysis from individual *white*$^+$ mutants derived from the CS2- allele reveals that all selected *w*$^+$ individuals carry a frame restorative NHEJ allele, while all *w*$^-$ alleles are not frame-restorative.

Supplementary Figure S6

Targeted DSB at *yellow* does not affect allelic conversion at *white*

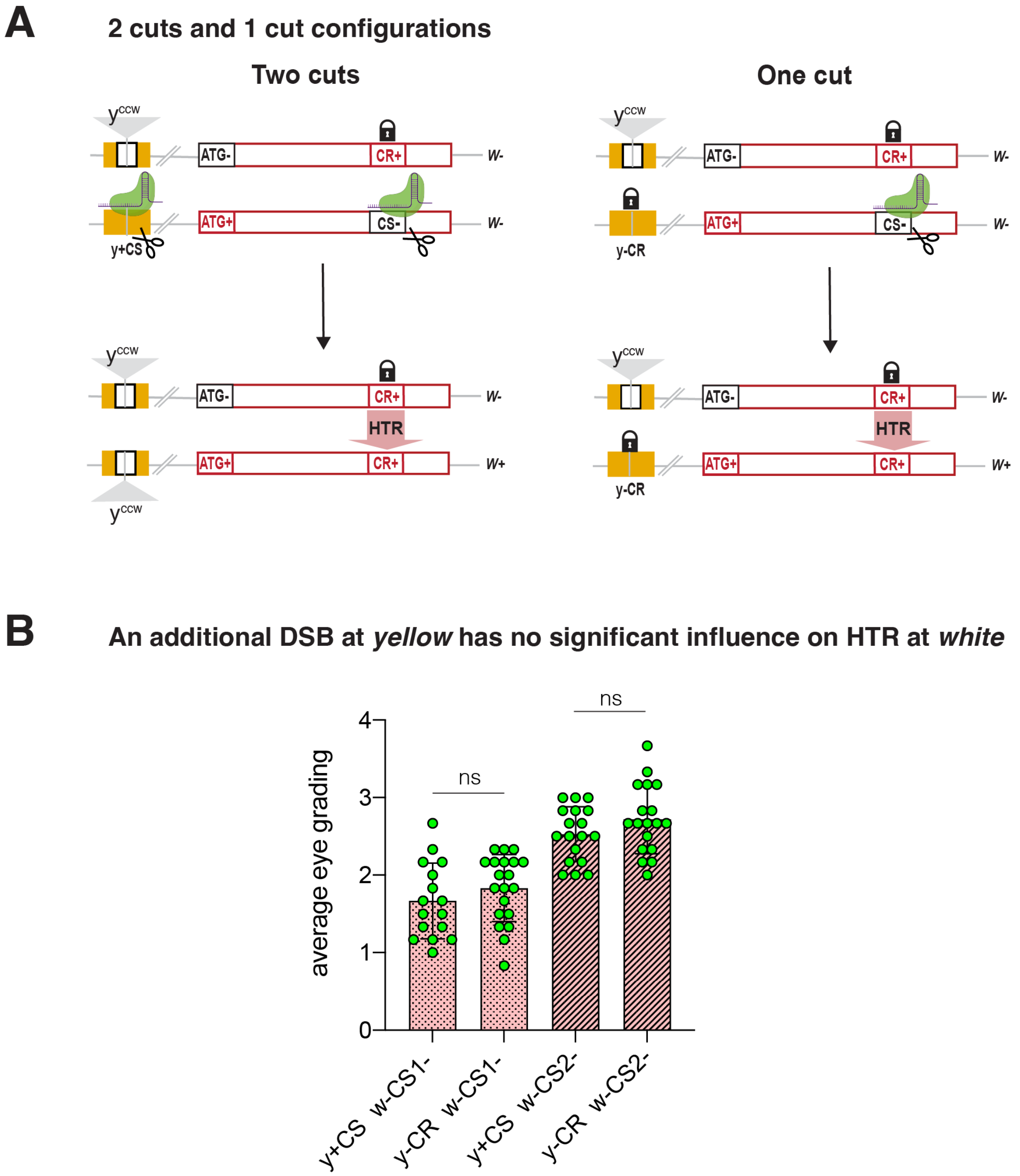

Fig. S6. Targeted DSB at *yellow* does not affect allelic conversion at *white*.

(A) Two-cut and one-cut configurations are used to evaluate the effect of targeted DSB at *yellow* (*y*) on allelic conversion (HTR) at *white* (*w*) in somatic tissues. The transgenic y^{ccw} element inserted in *y* encodes two guide RNAs, one targeting *y* at the site of its insertion, and the other (*white*-

gRNA), targeting cleavage at *w* (see Figure 1 and Materials and Methods). In the two-cut configuration, the *yellow*-gRNA directs cleavage of the cut-sensitive *y* wt allele (y^{CS+}) and the *white*-gRNA cleaves *w* near the CS1- or CS2- allele. In the one-cut configuration, a cut-resistant *y* allele (y^{CR+}, a ~2 Kb deletion) was used to prevent cutting, and only cleavage at *w* occurs. Successful repairs in both configurations produce visible red ($white^{+}$) eye clones. **(B)** Quantitative comparison of Cas9-induced allelic repair events in two-cut and one-cut configurations by eye grading analysis. For each configuration, eyes were ranked on a 0-5 scale and results from six eyes were averaged and plotted (see Figure S3). y^{CS+} $w^{ATG-\ CR+/ATG+\ CS1-}$ and y^{CR-} $w^{ATG-\ CR+/ATG+\ CS1-}$ flies show no significant difference in repair. Similarly, no significant difference was detected between y^{+CS} y^{CS+} $w^{ATG-\ CR+/ATG+\ CS2-}$ and y^{CR-} $w^{ATG-\ CR+/ATG+\ CS2-}$ flies , indicating that the additional DSB at *yellow* has no significant influence on HTR not NHEJ at *w*.

Supplementary Figure 7

Different repair phenotypes following DSB (Cas9) and SSB (D10A) revealed by GFP fluorescence

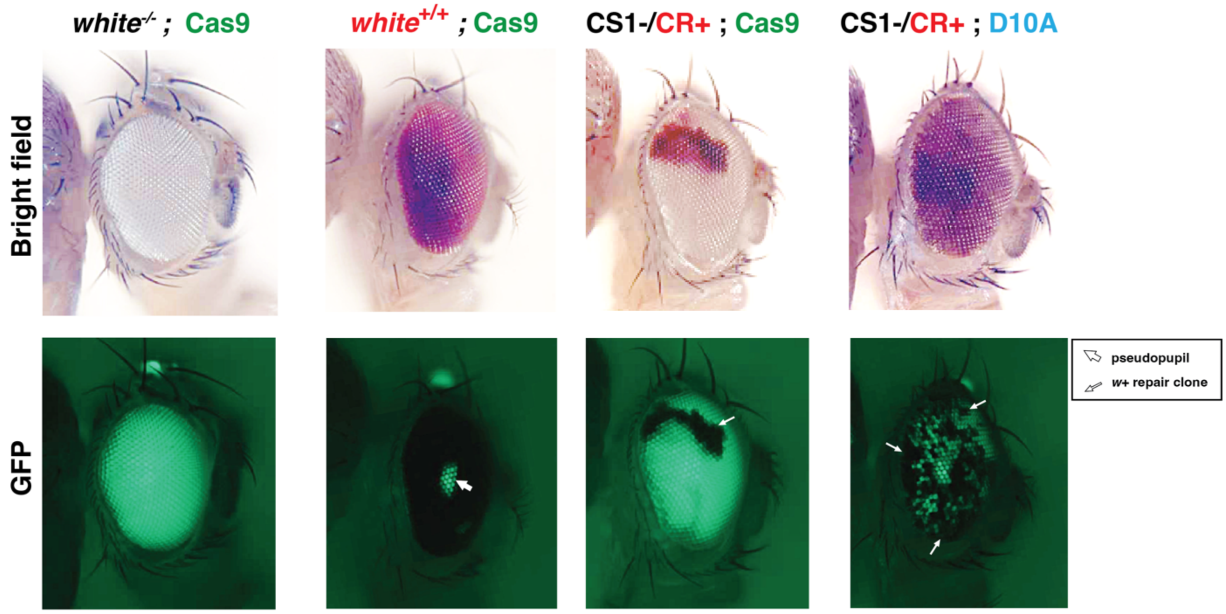

Fig. S7. Different repair phenotypes following DSB (by Cas9) and SSB (by D10A) revealed by white light and GFP fluorescence.

Top row shows bright-field images of eye phenotypes, bottom row shows GFP fluorescence images of the same eyes showing details of pigmentation phenotypes (green fluorescence originates from the 3XP3-GFP marker of vasaCas9 and vasaD10A insertions). Genotypes from left to right: $w^{-/-}$, $w^{+/+}$, y^{ccw} $w^{ATG-\ CR+}$/ $w^{ATG+\ CS1-}$; Cas9/+, and y^{ccw} $w^{ATG-\ CR+}$/ $w^{ATG+\ CS1-}$; D10A/+. In *w*- individuals, GFP fluorescence is visible throughout the eye. In w^{+} eyes, red pigments block the GFP fluorescence, except in the pseudo-pupil, where light shines through a group of ommatidia aligning with the observation axis (thick arrow). For y^{ccw} $w^{ATG-\ CR+}$ / $w^{ATG+\ CS1-}$; Cas9/+, and y^{ccw} $w^{ATG-\ CR+}$ / $w^{ATG+\ CS1-}$; D10A/+ individuals, in which w^{+} clones are produced through somatic HTR, GFP fluorescence is blocked by eye pigments, revealing the precise contours of each clone (thin arrows). For D10A-generated clones, this imaging reveals a dense array of small clones, while white light only reveals a more uniform and hazy pigmentation pattern.

Supplementary Figure 8

D10A elicits germline HDR/NHEJ events far less efficently than Cas9

A **Female germline HDR events revealed in F2 progeny**

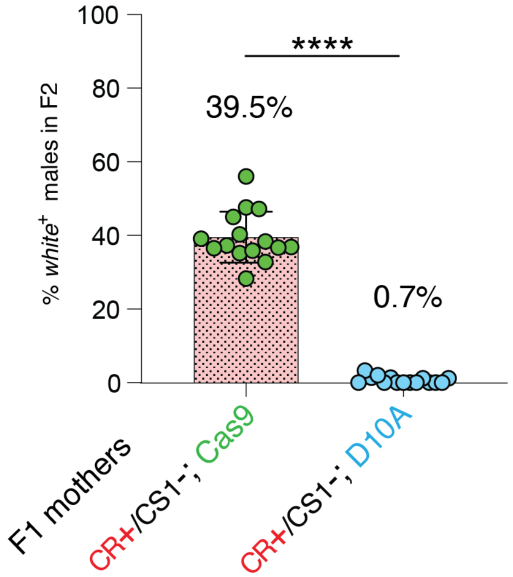

B **Male germline NHEJ events revealed in F2 progeny**

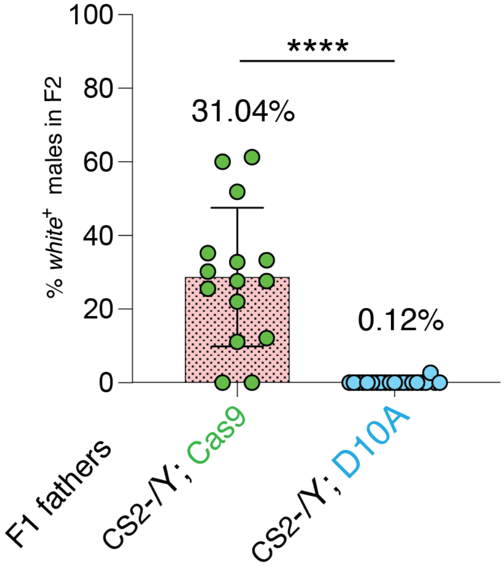

Fig. S8. D10A elicits germline HDR and NHEJ events far less efficiently than Cas9.

(A) HDR events in the germline of F1 y^{ccw} $w^{ATG- \ CR+}$/y^{+} $w^{ATG+ \ CS1-}$ females is revealed in F2 progeny by percentage of w^{+} males. D10A-induced HDR is very low (0.7%) comparatively to Cas9-induced HDR (39.5%). **(B)** NHEJ events estimated through percentage of F2 w^{+} male progeny from y^{ccw} $w^{ATG+ \ CS2-}$/Y; Cas9 or $w^{ATG+ \ CS2-}$/Y; D10A F1 males crossed to X^X females (see Fig. S5). While Cas9 induces high levels of NHEJ events (31%), D10A induces only 0.12% of NHEJ events.

Supplementary Figure 9

Deep-sequencing analysis of HDR and NHEJ events

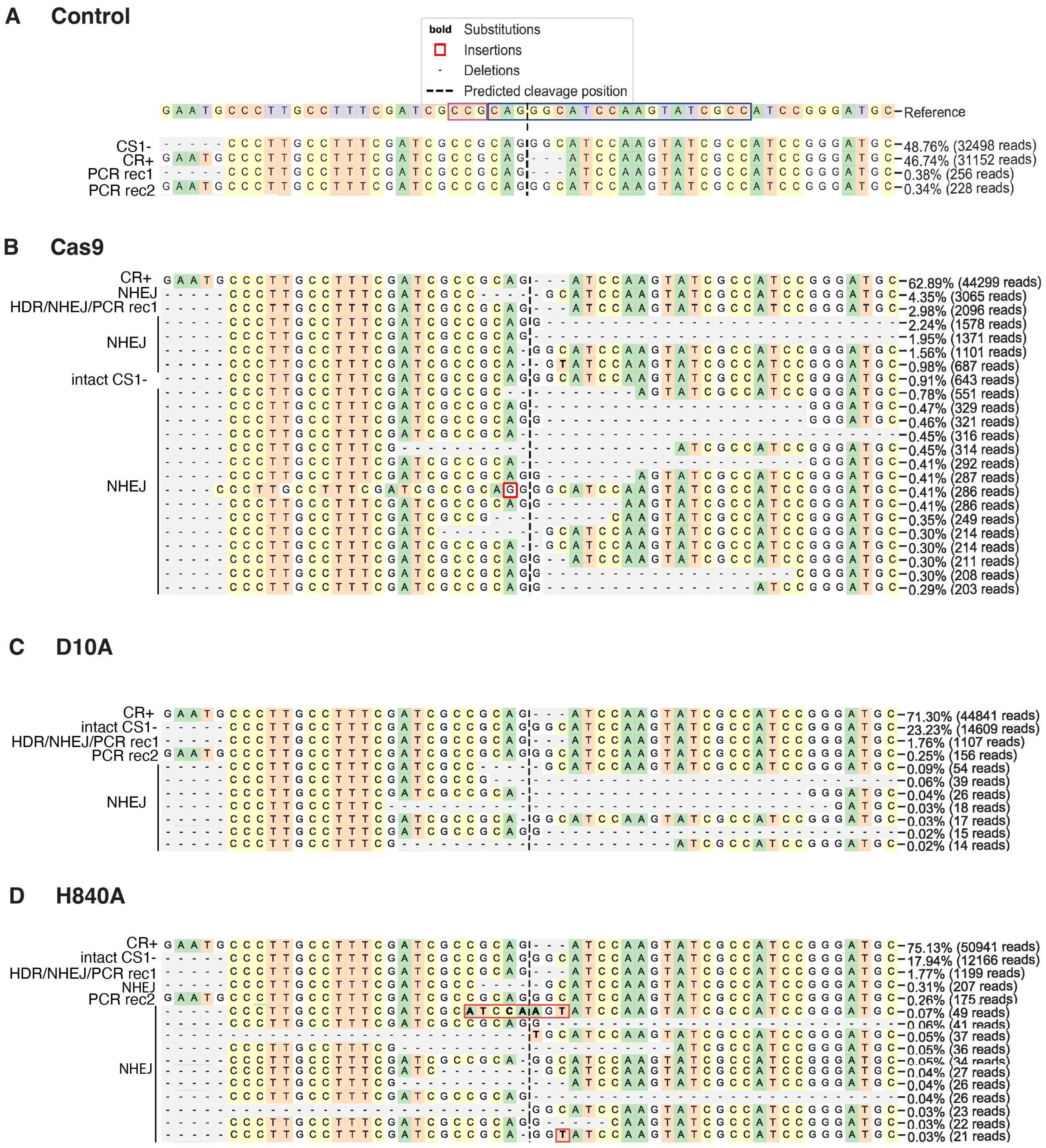

Fig. S9. Deep-sequencing analysis of HDR and NHEJ events.

(A) Deep-sequencing analysis of control y^{ccw} $w^{ATG- \ CR+}$/ y^{+} $w^{ATG+ \ CS1-}$ flies showing nearly equal percentage of CS1- and CR+ alleles. Other reads include PCR-induced recombination between the CS1- and CR+ mutations (0.38% for PCR recombination 1 and 0.34% for PCR recombination 2)

and substitutions (a total of 3.8%, not shown to preserve clarity). Sequences are aligned against the *white*$^{+}$ reference sequence, and the cut site is marked with a dotted line. For this panel and below, reads representing 0.02% and less were not shown. **(B)** Deep-sequencing analysis of *y*^{ccw} *w*$^{ATG-\ CR+}$/ *y*^{Cas9} *w*$^{ATG+\ CS1-}$ females reveals an elevated (62.9%) percentage of CR+ alleles, and a total of 33.2% mutations centered at the cut-site, corresponding to different NHEJ alleles. Intact CS1- alleles represent only 0.91%, indicating that most cut-sensitive sequences have been acted on. Association between CR+ and CS1- alleles amounts to ~3% of the reads (versus 0.38% in the control), which may include 1) HTR where the mismatch repair corrected heteroduplex DNA 3' to the CS1- allele , 2) *de novo* production of the CR+ allele through NHEJ and 3) PCR-induced recombination 1. **(C)** Deep sequencing analysis of D10A-expressing *y*^{ccw} *w*$^{ATG-CR+}$/*y*^{D10A} *w*$^{ATG+\ CS1-}$ flies revealed a higher proportion (71.30%) of CR+ alleles confirming that D10A elicits more efficient allelic conversion from CS1- to CR+ than Cas9. Only 0.35% of NHEJ alleles are detected. PCR- and cleavage-induced association between CR+ and CS1- alleles represent 1.76% of the reads (indicated as HDR/NHEJ/PCR rec1), while PCR recombination 2 represents 0.25%. Intact CS1- alleles represent ~23% of the reads. NHEJ deletions represent a calculated total 0.35% of total reads and tend to extend to the 3' side of the cut site. **(D)** Deep-sequencing analysis of H840A-expressing females (*y*^{ccw} *w*$^{ATG-CR+}$/*y*^{H840A} *w*$^{ATG+\ CS1-}$) revealed an even higher proportion (75.13%) of CR+ alleles, and a low proportion (0.42%) of NHEJ alleles, which extend to both 3' and 5' sides of the cut site. PCR- and cleavage-induced association between CR+ and CS1- alleles represent 1.77%, while PCR recombination 2 represents 0.26%. Intact CS1- alleles represent 17.94% of total reads.

Supplementary Fig 10

Tissue-specific HTR profiling: D10A-induced (but not Cas9-induced) allelic correction correlates with elevated *white* expression in the digestive and secretory systems.

CR+/CS1-

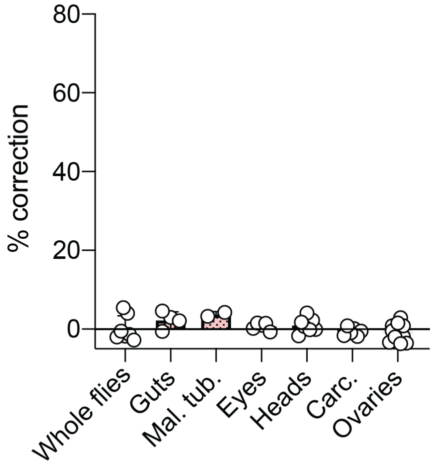

CR+/CS1- ; ; Cas9

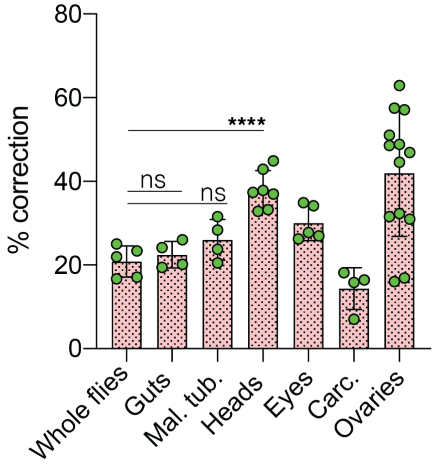

CR+/CS1- ; D10A

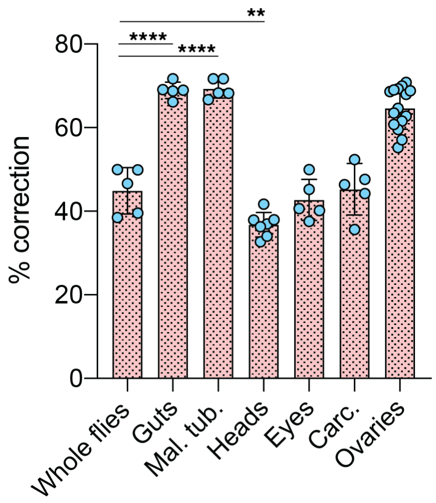

Fig. S10. HTR tissue-profiling: D10A-induced (but not Cas9-induced) allelic correction correlates with elevated *white* expression in the digestive and secretory systems.

Quantitative comparison of HTR in different tissues by Sanger sequencing. Correction percentages are calculated for dissected organs from 4-5 individuals using 9 peaks from electrophoretograms (peak 1-6, and 10-12, where peak 1 marks the first nt on the 5' end of the CS1- deletion, see Fig 2). No significant correction is visible in control y^{ccw}; $w^{ATG-\ CR+/CS1-}$ animals (no nuclease). In y^{ccw} $w^{ATG-\ CR+/CS1-}$; Cas9 flies, highest correction is observed in the ovaries (40%) followed by heads (37%) and then eyes (30%), lowest correction is observed in carcasses (17%), while whole flies , gut and malpighian tubules show similar correction (18-28%) . D10A flies (y^{ccw} $w^{ATG-\ CR+/CS1-}$; D10A) display higher overall correction (~45%) compared to Cas9 (~21%). Significantly elevated correction is observed in guts and Malpighian tubules (70%) and the ovaries (67%), while lowest correction is observed in heads (38%).

Supplementary Figure 11

D10A elicits somatic gene conversion (SGC), but less effciently than Cas9

A Copy-catchers reveal SGC through mutant phenptypes and DsRed expression

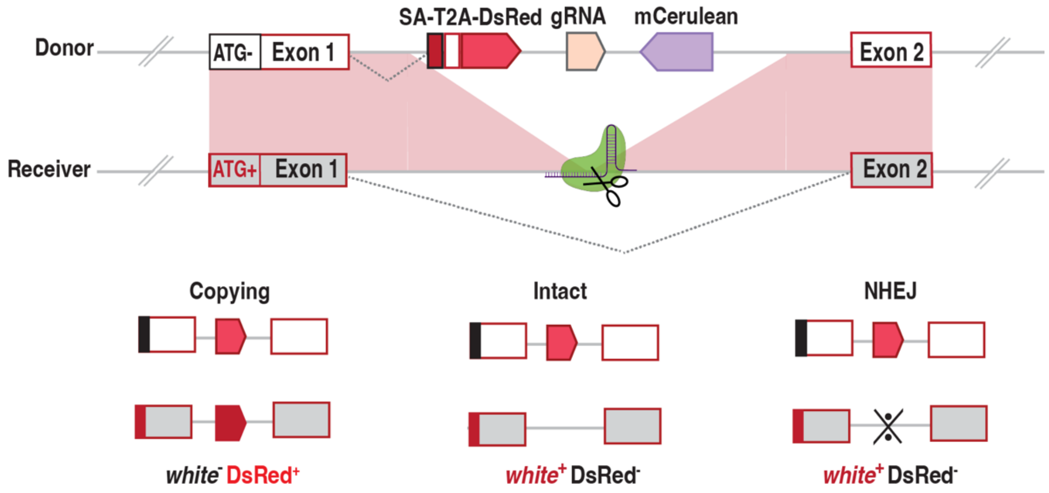

B Cas9 and D10A elicit different SGC patterns revealed by the *white* copy-catcher

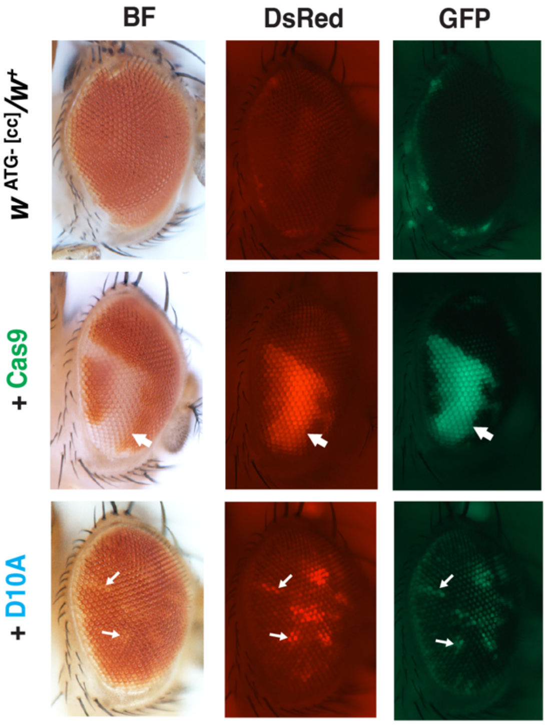

C *white* copy-catcher copying quantifications

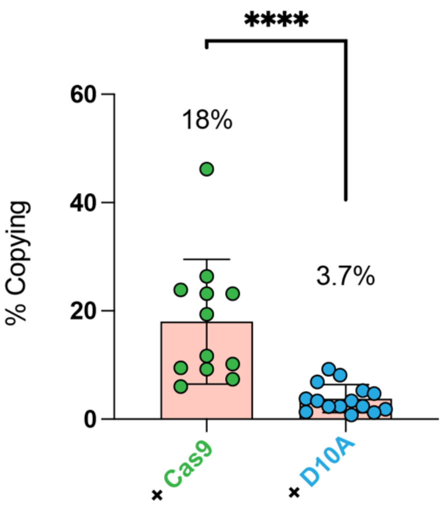

Fig. S11. D10A elicits somatic gene conversion (SGC), but less efficiently than Cas9.

(A) Diagram depicting how CopyCatcher insertions reveal somatic gene conversion (SGC,(*22*)). The $w^{[CC]}$ DNA cassette is inserted in intron 1 of *white* and introduces a splice acceptor site, which interrupts white coding sequences and creates a *white*$^{-}$ mutant allele. This cassette also includes a DsRed fluorescent marker in frame with the N-terminal *white* sequences, which is associated with an ATG- mutation in the donor chromosome to prevent expression. It also contains a gRNA promoting DNA cleavage on the naïve homologous chromosome at the insertion site of the $w^{[CC]}$ element. Upon targeted Cas9-dependent cleavage, inter-homologous DNA repair results in copying of the CopyCatcher onto the naïve chromosome, resulting in a homozygous l-o-f mutation with a visible *white*$^{-}$ clonal phenotype, and expression of the DsRed reporter (upon association with the ATG+ start codon). **(B)** Control $w^{[CC]/}$w+ heterozygous animals show red eyes and no DsRed expression. In Cas9-expressing $w^{[CC]/}$w+ females (middle row), large solid *white*$^{-}$ mutant eye clones (left panels) also expressing DsRed (middle column) reflect on successful copying events, while NHEJ events (affecting only intronic sequences) remain phenotypically silent. GFP fluorescence (expressed from the vasaCas9 insertion) also shines through the unpigmented *white*$^{-}$ tissues (right-side panels). In $w^{[CC]/}$w+; vasaD10A animals, few small *white*- w[CC]/w[CC] DsRed+ clones are visible (thin arrows) indicative of successful gene conversion. **(C)** Quantification of SGC as evaluated by the percentage of total GFP+ areas on individual eyes. Cas9 elicits high levels of SGC (18%), while D10A is 4-5 times less efficient (3.7%).

Supplementary Figure 12

Three different P(*white*$^+$) insertions provide template for pairing-independent allelic repair

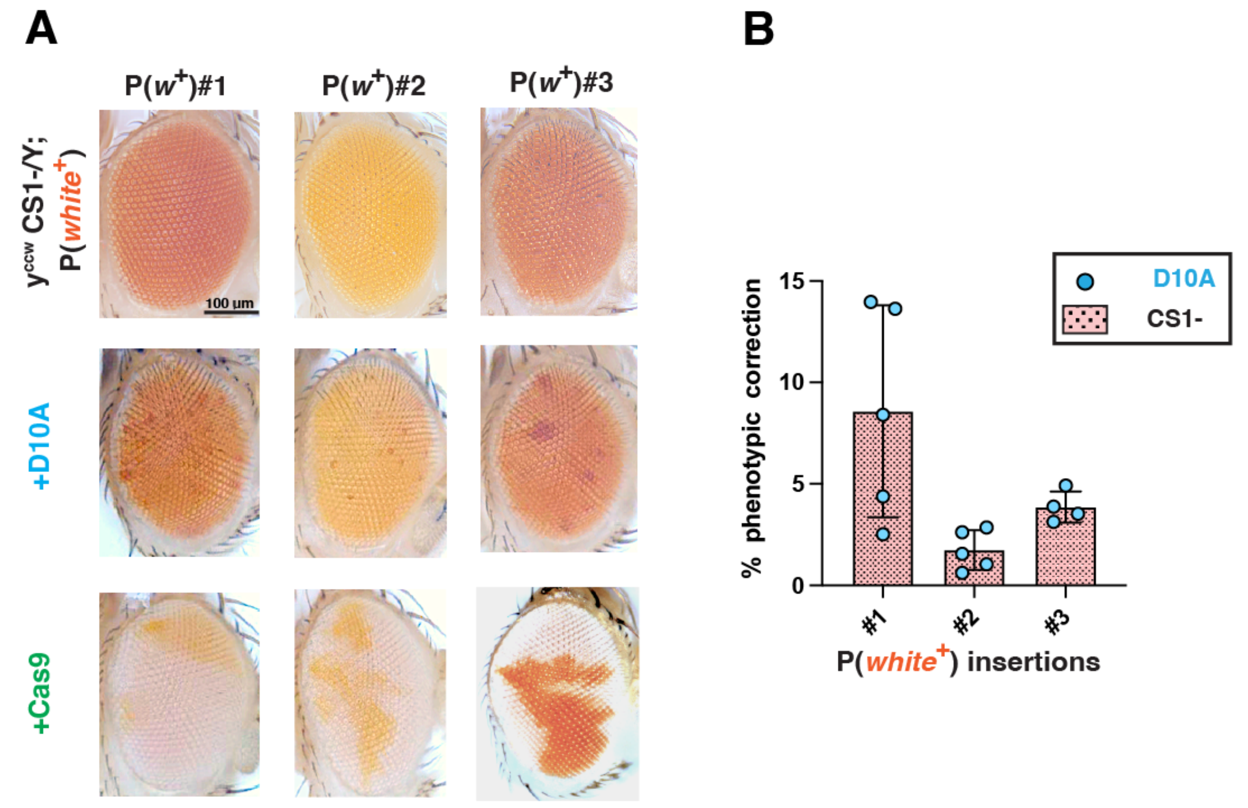

Fig. S12. Pairing-independent allelic repair.

(A) Eyes from y^{ccw} $w^{ATG+\ CS1-}$/ Y males carrying the following P(*white*$^+$) autosomal insertions: #1: P{GAL4-Hsp70.PB}(*34*)89-2-1 (BL#1799, 3rd chromosome, first column); #2: P{GAL4-Hsp70.PB}2 (BL#2077, 2nd chromosome, second column), #3: P{GAL4-Hsp70.PB}31-1 (BL#1822, 3rd chromosome, third column). Top row: eyes from y^{ccw} $w^{ATG+\ CS1-}$ / Y; P(*white*$^+$)/+ control males. Eye color (orange or yellow) is specific to each P(*white*$^+$) insertion. Second row: eyes from y^{ccw} $w^{ATG+\ CS1-}$ / Y ; P(*white*$^+$)/+ males expressing D10A. Red clones indicate that the CS1- allele was corrected by SAC using wild type sequences provided by the P(*white*$^+$) autosomal insertion. Background orange eye color tends to be lighter in these D10A animals than in corresponding controls, suggesting that gRNA complexes binding to the targeted sequence affect transcription efficiency. Third row: eyes from y^{ccw} $w^{ATG+\ CS1-}$ / Y ; P(*white*$^+$)/+ males expressing Cas9. Large white clones indicate that NHEJ-dependent mutations were produced in the P(*white*$^+$)

insertion. No red clone was observed, indicating that Cas9 did not elicit pairing-independent allelic conversion. **(B)** Image-based quantification of pairing-independent repair of the CS1- allele for each P(*white*$^{+}$) autosomal template insertion. Different repair rates suggest that chromosomal position, transcriptional activity and other factors influence this process.
